# Meta-Analysis of Abiotic Conditions Affecting Exopolysaccharide Production in Cyanobacteria

**DOI:** 10.3390/metabo15020131

**Published:** 2025-02-14

**Authors:** Shijie Wu, Fuwen Wang, Hong Wang, Cong Shen, Kaiqiang Yu

**Affiliations:** 1 College of Resources and Environment and Life Sciences, Ningxia Normal University, Guyuan 756000, China; wushijie1128@163.com (S.W.); wh12100912@163.com (H.W.); shencong0226@163.com (C.S.); 2 Key Laboratory of Soil Ecological Health and Microbial Regulation, Ningxia Normal University, Guyuan 756000, China; 3 Research Center for Eco-Environmental Sciences, Chinese Academy of Sciences, Beijing 100085, China; wangfuwen231@mails.ucas.ac.cn

**Keywords:** meta-analysis, cyanobacteria, exopolysaccharides, abiotic conditions, genus specificity

## Abstract

**Background**: cyanobacterial exopolysaccharides (EPSs) exhibit diverse biological and physicochemical properties, making them valuable for applications in environmental remediation, soil improvement, wastewater treatment, and bioenergy production. **Results**: the production of cyanobacterial EPSs is significantly influenced by various factors, including abiotic factors and strains. Recent research has focused on optimizing EPS production by regulating key abiotic factors such as light, temperature, pH, and nutritional conditions. This review systematically compiles and analyzes published data on the effects of abiotic factors on cyanobacterial EPS biosynthesis, with a focus on genus-specific responses. Using meta-analysis techniques, we provide a comprehensive overview of the key factors influencing EPS production. Light and nutrient conditions are the most significant factors affecting EPS production, with high light intensities and optimal nutrient conditions enhancing EPS synthesis. Optimal temperature ranges and pH levels are essential for maximizing EPS production, and cyanobacteria exhibit genus-specific responses to variations in these factors. The addition of specific nutrients, such as NaCl, trace metals (e.g., Mg, Zn, Cu), and elevated CO_2_ levels, significantly impacts EPS production. **Conclusions**: the response to these factors varies among different cyanobacterial genera, highlighting the need for genus-specific optimization strategies. This review provides a theoretical basis for optimizing EPS production across diverse cyanobacterial genera and for understanding multi-factor interactions and practical applications in future research.

## 1. Introduction

Cyanobacteria, as a large and widespread group of photoautotrophic microorganisms, are able to perform oxygenic photosynthesis [1]. Cyanobacteria have a potential application on the sustainable agriculture and environmentally safe, which is due to their excellent nitrogen and carbon fixation abilities [2]. Exopolysaccharides (EPSs) are complex polymeric carbohydrates which are present around their cells as an enveloped layer and which are released outside [3]. EPSs also perform a variety of physicochemical and potentially biological properties [4], such as improving soil physicochemical properties and fighting against environmental stress through the physiological and ecological functions of EPSs [5,6]. Remarkably, cyanobacteria, which are a major source of commercialized EPSs, have EPSs surrounding their cells [7]. In recent decades, many studies have been carried out on the EPS applications of cyanobacteria [3,8]. Scientists have discovered that cyanobacterial EPSs have important physiological significance and mainly play a protective role against various stresses (e.g., ultraviolet radiation, drought, freezing) [9,10,11,12]. Moreover, cyanobacterial EPSs also perform a huge range of functions, including soil stabilization, antioxidants, moisture absorption and retention, carbon sinks, structural modification, dust trapping, and the recovery of heavy metals from wastewater [13,14,15,16,17,18]. In addition, cyanobacterial EPSs have been reported to possess the antitumor, antioxidant, and antiviral properties [19]. Consequently, the production of cyanobacterial EPSs is of increasing interest in the science community.

Cyanobacterial EPS production is significantly affected by many factors such as culture conditions and strains. It is worth noting that climate change is a critical problem not only for animals, plants, and large algae, but also cyanobacteria often face the same issues. The results of global warming, such as temperature rises, droughts, and increased carbon dioxide excess, affect cyanobacterial EPS production. Our study is an elaborate review of cyanobacterial EPSs, aiming to probe their possible relationship between above factors and cyanobacterial EPS. A better selection of cyanobacterial EPS production parameters and species is very important to develop a cyanobacterial EPS bioprocess. And understanding the factors of EPS production in cyanobacteria is of great value for exploring the impact of EPSs on the natural community, particularly sustainable agriculture, algal bloom, and wastewater treatment.

In this review, we systematically compiled and analyzed published data on the effects of abiotic factors on cyanobacterial EPS biosynthesis, with a focus on organizing findings at the genus level. All figures in the manuscript are based on raw EPS data from Table S2 (Supplementary Materials), with fold changes calculated by dividing treatment group EPS values by those of the control group. These fold change values were used to generate the manuscript figures, ensuring a consistent and comparative analysis across studies. Using meta-analytic techniques, we pooled effect sizes from individual studies to provide a new overview of the key factors influencing EPS biosynthesis, offering a novel perspective that emphasizes genus-specific responses. By investigating the influence of abiotic factors on EPS content across different cyanobacterial genera, critical insights into their adaptive mechanisms under diverse environmental conditions can be obtained. A deeper understanding of these mechanisms is essential for elucidating the ecological roles of cyanobacteria. Furthermore, this knowledge provides a solid scientific foundation for developing effective strategies to manage cyanobacterial blooms, restore ecosystems, and advance biotechnological applications.

## 2. Cultivation Medium for Cyanobacteria

The data in Table 1 are derived from the references listed in Table S1 (Supplementary Materials). The cultivation of cyanobacteria can be conducted by diversifying growth conditions, depending on the species, such as BG11, Alkaline, F/2, Zarrouk, YBCII, and others, as shown in Table S1 and Table 1. They each have their own characteristics, specifically designed for the cultivation of different microorganisms or under specific conditions.

BG-11 medium, also recognized as the cyanobacteria medium, stands as a highly preferred cultivation medium tailored for freshwater algae, protozoa, and notably cyanobacteria species [20,21,22,23,24]. From the data in Table 1, it is evident that the majority of the cyanobacterial strains demonstrate an ability to grow in BG-11 medium. Since some cyanobacteria possess the ability of nitrogen fixation, in order to study this process, some culture mediums utilize BG-11 medium without the addition of NaNO_3_. Alkaline is suitable for microorganisms that thrive in alkaline environments. It is commonly used to study the growth characteristics and metabolic activities of microorganisms under various pH conditions. F/2 medium is a commonly used culture medium for cultivating unicellular algae, such as *Synechocystis*. Zarrouk medium is commonly used for the cultivation of *Spirulina* [25,26]. It has a high pH value and a high concentration of sodium bicarbonate (NaHCO_3_). YBC-II medium is an artificial seawater medium, also known as the *Trichodesmium* medium. All these media contain the similar nutritional elements but with slight modifications.

## 3. Effect of Abiotic Conditions for Cyanobacterial EPS Production

**Figure 1:**
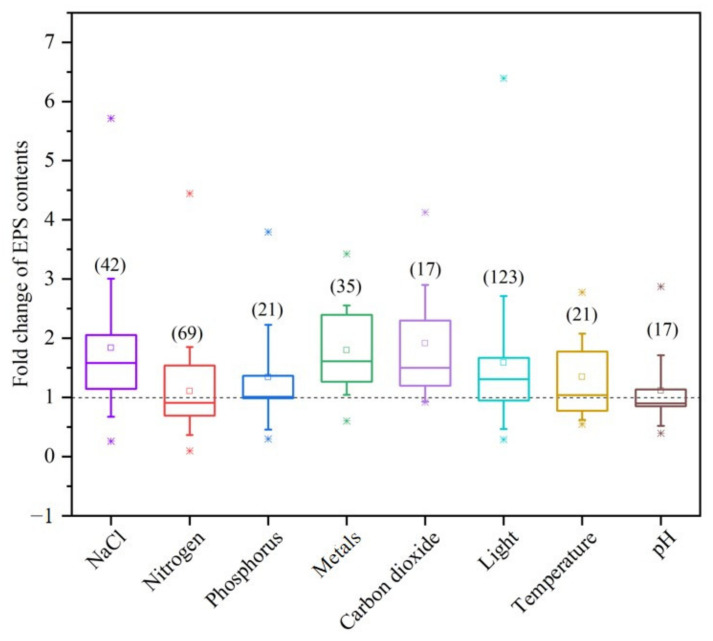
Fold change in EPS content variability according to the operating conditions such as NaCl, nitrogen, phosphorus, metals, carbon dioxide, light, temperature, and pH. The data were extracted from 45 publications (each publication could contain several values depending on the tested conditions). The box plots include the medians (lines) and the means (squares). The asterisks indicate the highest and least values encountered for each condition.

In the past few decades, researchers have conducted increasingly in-depth studies on the production of cyanobacterial EPSs by regulating several key abiotic factors, with the aim of elucidating the mechanisms of EPS formation in cyanobacteria and exploring its industrial applications [4]. This study employs a meta-analysis to investigate the effects of abiotic factors on the content of EPSs in cyanobacteria, aiming to identify the key abiotic factors that regulate EPS synthesis. In this view, all the EPS contents of cyanobacteria available in the literature are depicted in Figure 1 and gathered in the Table S1. These EPS content values were primarily categorized and collected based on various growth conditions, encompassing nutritional factors such as NaCl, nitrogen, phosphorus, metals, and carbon dioxide, as well as environmental parameters including light, temperature, and pH levels.

As depicted in Figure 1, light as the predominant abiotic factor is investigated in the context of cyanobacterial EPS research. Notably, light and nitrogen emerge as the most influential factors, with light intensity and quality directly impacting photosynthetic efficiency and carbon fixation, while nitrogen availability modulates the carbon-to-nitrogen ratio, a critical determinant of EPS production. As a category of photosynthetically autotrophic prokaryotes, cyanobacteria mainly depend on the carbon sources produced during photosynthesis for the biosynthesis of their EPS. Light, serving as the energy source for photosynthesis, has a decisive effect on the synthesis of cyanobacterial EPSs. Therefore, a substantial amount of research has been conducted by scholars to explore how light, an abiotic factor, affects the synthesis of cyanobacterial EPSs, with the goal of improving the yield and quality of cyanobacterial EPSs by optimizing light conditions. This selection process resulted in a collection of 123 datasets encompassing the impact of light on cyanobacterial EPS synthesis, as reported in 27 scholarly publications (Figure 1). Concurrently, in our assessment of the impacts of macro-nutrients, the datasets encompassed 69 distinct values derived from an extensive review of 30 publications pertaining to nitrogen. Furthermore, a compilation of 21 datasets harvested from eight publications shed light on the role of phosphorus. Within the trace metal category, which included calcium, magnesium, copper, and zinc, our analysis incorporated data gleaned from 35 values across eight scholarly publications. Additionally, the datasets for NaCl included 42 values extracted from 11 publications, further enriching our analysis of the nutritional and environmental factors influencing cyanobacterial EPS production. Meanwhile, a body of studies has investigated the impact of other abiotic factors, such as temperature, pH, and carbon dioxide, on the synthesis of cyanobacterial EPS. These investigations have elucidated the roles of these environmental variables in regulating the biosynthesis of cyanobacterial EPS, providing a scientific rationale for optimizing cultivation conditions to enhance their production. Generally, our results showed that the means of all growth conditions could increase the cyanobacterial EPS contents, mainly focusing on light, nitrogen, and NaCl conditions. Over three-quarters of values have been shown to increase the EPS content under metal, carbon dioxide, and NaCl addition operating conditions. And the studies have found light offers a higher ranges of EPS content than other growth conditions (Figure 1). In conclusion, our findings offer significant perspectives on the abiotic factors affecting EPS production in cyanobacteria. By understanding the impact of these factors, we can optimize growth conditions to maximize EPS production for various applications. Future research should focus on elucidating the molecular mechanisms underlying EPS synthesis and secretion in cyanobacteria and exploring the potential use of these organisms in sustainable bio-energy production and other industrial processes.

### 3.1. Effect of Light Conditions for Cyanobacterial EPS Production

**Figure 2:**
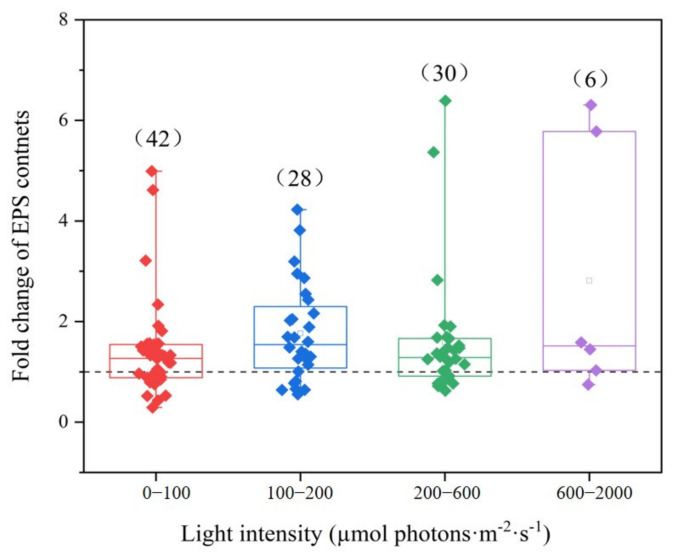
Fold change in EPS content variability according to different light intensities. Each dot represents a specific value. The box plots include the medians (lines) and the means (squares).

Light indirectly affects the synthesis of EPS in cyanobacteria by regulating the efficiency of photosynthesis. Changes in light intensity, light quality (wavelength), and light exposure duration can significantly influence the yield and composition of EPSs [27,28]. To evaluate the effect of the light intensity to EPS contents, the data were classified in four categories (Figure 2). The majority of light values were focused with 0 to 600 µmol photons·m^−2^·s^−1^, as shown for *Anabaena* sp. ATCC 33047 [29], *Spirulina platensis* [30], *Arthrospira platensis* [31], *Cyanothece* sp. 113 [32], *Gloeocapsa gelatinosa* [33], *Nostoc* sp. PCC 8113, PCC 7936, PCC 7413 [34], *Nostoc flagelliforme* [35], *Nostoc* sp. [36], *Microcoleus vaginatus* [37], *Scytonema tolypothrichoides* and *Tolypothrix bouteillei* [38], *Cyanobacterium aponinum* PCC 10605 [39], and *Microcystis aeruginosa* [40,41,42]. Only a few studies have looked at higher light intensities (600 and 2000 µmol photons·m^−2^·s^−1^). For example, Moreno et al. [29] studied cyanobacterial EPS production with photobioreactor to culture. Using cultivating containers (shake-flask or photobioreactor), even culture volume, it seems that field experiments could demand a remarkably higher light intensity than in laboratories, suggesting that we should be concerned with differences in cyanobacterial EPS production via both field experiment and laboratory research in order to provide a better scientific basis for practical application. EPS production often increases under stress [43]. Notably, ultraviolet B (UV-B) radiation functions as an environmental stressor that markedly influences the photosynthetic processes and growth dynamics of cyanobacteria. Within this framework, EPSs released by cyanobacteria assume a critical role. These substances not only confer protection against the detrimental effects of UV-B radiation but also enhance the cyanobacteria’s capacity to adapt to the environmental pressures triggered by UV-B [44]. Researchers found that UV-B radiation stimulated cyanobacterial EPS production in *Nostoc punctiforme* ATCC 29133 and scytonemin-deficient mutant, SCY59 [45], *Nostoc flagelliforme* [12], and *Microcystis aeruginosa* [42]. Moreover, light quality significantly influences the production of EPSs in cyanobacteria. There is research indicating that varying light quality conditions, including different wavelengths and varying intensities, can modify the metabolic pathways within cyanobacteria, consequently impacting the synthesis and accumulation of EPSs [10,46]. Existing research indicates that light quality (e.g., red, blue, green, yellow, purple, white, etc.) influences the production of EPSs in cyanobacteria, with a predominant focus on terrestrial species. In contrast, studies examining the impact of light quality on aquatic cyanobacteria are relatively scarce, necessitating further investigation into how different light qualities affect EPS production in these organisms [11,12,46,47]. Light quality profoundly influences the biosynthesis of cyanobacterial EPSs, serving as a protective shield for cyanobacteria against UV-B radiation. It also plays a crucial role in regulating photosynthesis and metabolic pathways, thereby affecting the synthesis and accumulation of EPSs. Further exploration of the underlying molecular mechanisms is essential to establish a foundation for optimizing EPS production in the future.

### 3.2. Effect of Temperature and pH Conditions for Cyanobacterial EPS Production

**Figure 3:**
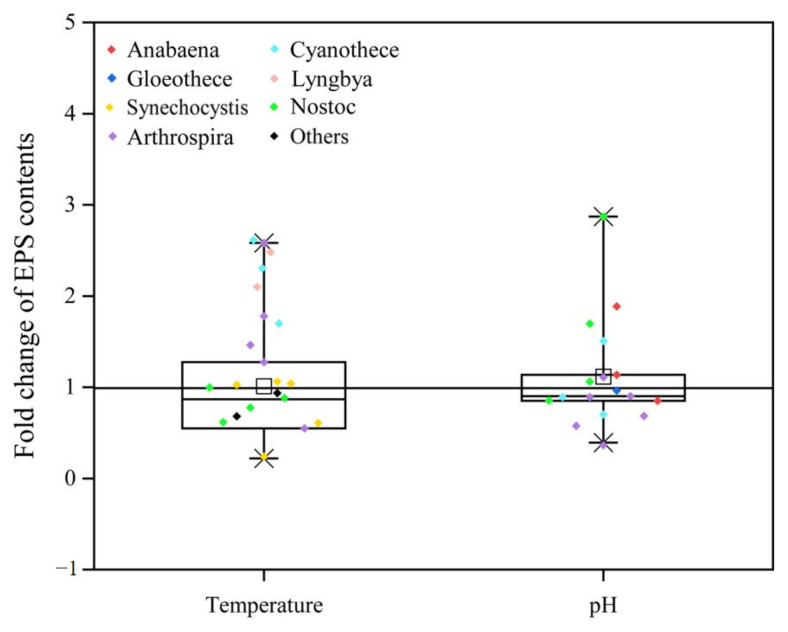
Fold change in EPS content variability according to temperature and pH. Each dot represents a specific value, with different colors indicating different cyanobacterial genera. The box plots include the medians (lines) and the means (squares).

Temperature significantly impacts cyanobacterial growth and metabolism by modulating enzymatic activity, which in turn affects EPS synthesis and cellular integrity. Optimal temperatures enhance EPS production, while extreme temperatures can inhibit it, leading to cyanobacterial stress or death. The study of growth temperature variation on cyanobacterial EPSs has been conducted, demonstrating the genus specificity (Figure 3). For *Cyanothece* sp. 113, *Spirulina platensis*, *Lyngbya stagnina*, and *Microcystis aeruginosa* Kützing PCC 7806, an increase in the temperature led to a considerable increase in cyanobacterial EPS production [30,32,41,48]. In contrast, the EPS production had an opposite change with an increase in the temperature in *Nostoc* PCC 7936, *Cyanobacterium aponinum* PCC 10605 and *Microcystis aeruginosa* [39,41,49]. Higher temperature is a vital factor for growth and EPS production by cyanobacteria, probably because the time required to reach the onset of stationary phase is shorter at higher temperatures [4]. Furthermore, elevated temperatures could alter the chemical composition and properties of EPSs, including the length and degree of branching of polysaccharide chains, and also the structure of proteins.

In summary, the impact of temperature on cyanobacterial EPSs is multifaceted, encompassing effects on the growth and reproduction of cyanobacteria, the composition and content of EPSs, and the dewatering performance of EPSs. Therefore, in the process of cyanobacterial treatment, it is essential to fully consider the temperature factor and adopt appropriate measures to optimize treatment outcomes. In doing so, we can enhance the efficiency of cyanobacterial management and mitigate the potential negative impacts of temperature fluctuations on EPS properties and cyanobacterial activity.

In the process of EPS synthesis by cyanobacteria, alterations in pH significantly influence the metabolic activity of the microorganisms, thereby affecting the rate of EPS synthesis and the extent of accumulation. The results depicted in Figure 3 reveal that the genus *Nostoc* is more prone to synthesizing EPS in environments with higher pH values. If the pH values set in the treatment medium are lower than those in the original culture medium, the cyanobacterial EPS content decreased in the family Nostoc, and vice versa [50]. The same observation has been made for the cyanobacterial EPSs of *Nostoc flagelliforme* [51]. But, for *Spirulina platensis*, the pH in the original culture medium seemed to be the best condition to produce cyanobacterial EPSs [52]. In particular, the growth and EPS synthesis in aquatic cyanobacteria exhibit heightened sensitivity to subtle pH variations. These fluctuations can markedly influence cyanobacterial growth and EPS production through their effects on environmental acidity, carbonate equilibrium, and the partitioning of inorganic carbon species. Investigations have demonstrated that *Arthrospira platensis* is better suited to alkaline conditions, with growth and EPS accumulation being adversely affected under slightly acidic conditions (pH values below 7) (Figure 3). Furthermore, studies have indicated that while strong alkalinity suppresses the growth of *Arthrospira platensis*, it concurrently stimulates an increase in EPS secretion [53]. Research has found that a decrease in pH leads to an increase in the EPS content in *Trichodesmium erythraeum* [54,55]. Existing research commonly focuses on the impact of pH changes in the culture environment on the content of cyanobacterial EPSs. In fact, fluctuations in pH can also affect the solubility, mobility, and transformation of EPSs in environmental media. For instance, under acidic or alkaline conditions, EPSs may react with inorganic substances in the environment, leading to the formation of precipitates or complexes. These reactions can subsequently influence the distribution and fate of EPSs in the environment, thereby affecting the stability and functionality of ecosystems. Therefore, when studying and applying EPSs, it is necessary to fully consider the important factor of pH.

### 3.3. Effect of Nutritional Conditions for Cyanobacterial EPS Production

#### 3.3.1. Nitrogen and Phosphorus

**Figure 4:**
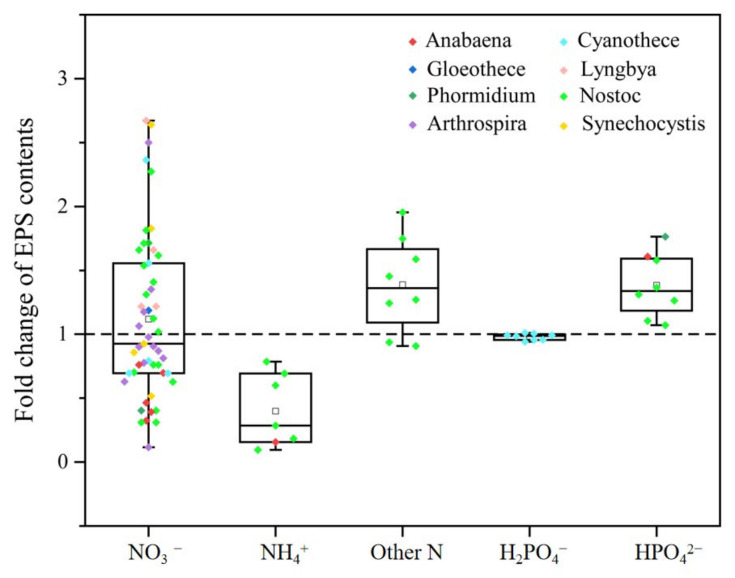
Fold change in EPS content variability according to the operating conditions such as NO_3_^−^, NH_4_^+^, other nitrogen sources (other N), HPO_4_^2−^, and H_2_PO_4_^−^. Each dot represents a specific value, with different colors indicating different cyanobacterial genera. The box plots include the medians (lines) and the means (squares).

Nitrogen is a paramount element of vital compounds (e.g., nucleic acid, protein, and pigment) and cyanobacteria are either dependent on fixing atmospheric nitrogen or combined nitrogen sources [4]. Many studies have explored the potential effects of different nitrogen sources on the growth and development of various cyanobacterial species (Table S1). Cyanobacterial EPS production may be affected by the chemical nature of nitrogen sources [28]. Regarding the available EPS content data summarized in Figure 4, the fold change in EPS contents can be observed, by adding nitrate (NO_3_^−^) to create conditions ranging from 0.12 to 2.67 and adding ammonium (NH_4_^+^) in the range from 0.10 to 0.79. In addition, other nitrogen sources (arginine, urea, peptone, and casein) have also been described as condition that enhanced EPS contents [11]. Among the different nitrogen sources, NO_3_^−^ was a quite extensive one for the production of EPS contents, mainly focusing on the genus *Nostoc*, *Synechocystis*, *Arthrospira*, *Gloeothece*, *Anabaena*, *Cyanothece*, *Lyngbya*, and *Phormidium* (Figure 4). Upon an analysis of Figure 4, it is observed that the production of cyanobacterial EPSs under NO_3_^−^ conditions exhibits notable complexity. Tease and Walker discovered that an increase in NO_3_^−^ levels led to a higher production of EPSs in the Cyanobacterium *Gloeothece* ATCC 27152 [56]. Upon the addition of NO_3_^−^, significant variability in the EPS content is observed even among cyanobacteria of the same genus. For instance, this disparity is particularly pronounced in the genus *Cyanothece*. In *Cyanothece* sp. 113, cyanobacterial EPS production decreased as the added NO_3_^−^ concentrations increased [32], but the opposite result was found in *Cyanothece* ATCC 51142 [57]. These results remind us to pay more attention to the reason for EPS production in the same genus in response to nitrogen limitation.

Under nitrogen-sufficient conditions, cyanobacteria of the *Nostoc* and *Synechocystis* exhibit high EPS contents, as illustrated in Figure 4. This high EPS production under ample nitrogen conditions is contrasted by findings from De Philippis et al. [58], who reported that nitrogen limitation can significantly enhance polysaccharide yields in *Cyanothece*. This enhancement under nitrogen limitation suggests that cyanobacteria have the metabolic flexibility to adapt to nitrogen-deficient environments by increasing EPS synthesis, which serves as a strategy to cope with environmental stress. The observed increase in EPS production under nitrogen limitation is likely due to the improvement in the carbon-to-nitrogen (C:N) ratio, which promotes more EPS synthesis, as noted by Otero and Vincenzini [34] and Kumar et al. [59]. However, upon the addition of nitrate, data from various studies indicate a trend of decreased EPS content in cyanobacteria. This decrease suggests that the modulation of EPS content in response to nitrate availability is a complex process, influenced by a multitude of environmental and physiological factors. The contrasting responses to nitrogen sufficiency and limitation highlight the intricate regulatory mechanisms within cyanobacteria, which fine-tune EPS production according to the nitrogen status of their environment.

Currently, research on the role of phosphorus in the synthesis of EPSs in cyanobacteria is relatively scarce, with a particular focus on the genus *Nostoc*. Phosphorus is also an essential macroelement in the growth of cyanobacteria; the correlation between phosphorus source and EPS contents has been evaluated for different cyanobacteria, and some results were observed depending on the genus studied [32,41,60,61,62]. Usually, as can be observed in Figure 4, by adding the hydrogen phosphate ion (HPO_4_^2−^) conditions, the cyanobacteria of the genus *Nostoc, Phormidium*, and *Anabaena* resulted in an increase in EPS production. Su et al. [32] showed that *Cyanothece* had no effects of added the dihydrogen phosphate ion (H_2_PO_4_^−^) on EPS production.

#### 3.3.2. NaCl, Metals, and Carbon Dioxide

**Figure 5:**
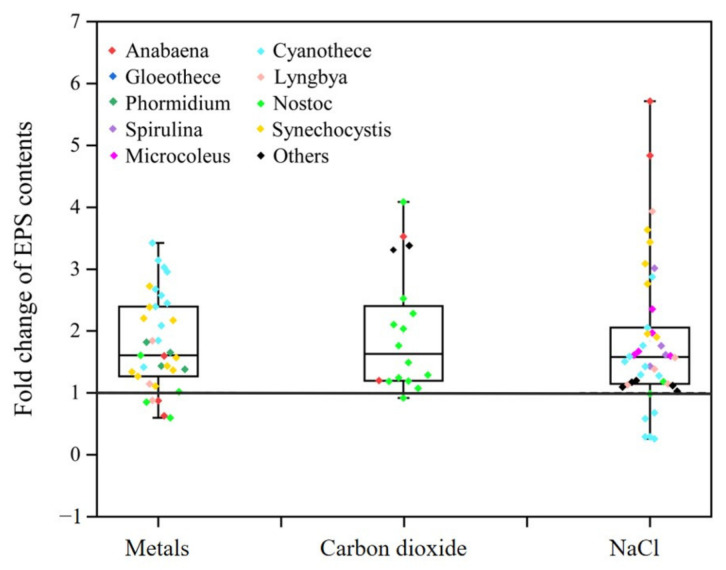
Fold change in EPS content variability according to the metals, carbon dioxide, and NaCl conditions. Each dot represents a specific value, with different colors indicating different cyanobacterial genera. The box plots include the medians (lines).

The influence of NaCl stress on the synthesis of EPSs in cyanobacteria has been the subject of increasing interest. Our analysis of the literature indicates that, in general, cyanobacterial cultures supplemented with NaCl tend to produce higher quantities of EPSs compared to those without NaCl supplementation (Figure 5). Furthermore, as the NaCl concentration increases, there is a significant rise in the EPS content of cyanobacteria, a pattern observed across various studies [57,63,64]. Given the diversity among different cyanobacterial strains, we suggest that future research should focus on determining the optimal NaCl concentration to maximize EPS production. Our findings reveal a strong inverse correlation between EPS content and cyanobacterial growth under NaCl stress conditions (Table S1). This correlation suggests that NaCl stress may act as a stimulus for cyanobacteria to increase EPS production, potentially as a protective mechanism for the cells. The modulation of EPS synthesis in response to NaCl stress in cyanobacteria is a significant area of research. Understanding these adaptive mechanisms is crucial for comprehending how cyanobacteria cope with high-salinity environments. Moreover, elucidating the role of EPSs in saline adaptation could unveil its industrial applications, particularly in the context of biofilm formation and bioprocesses where salinity is a critical factor. Therefore, further investigation into the interplay between NaCl stress and EPS synthesis in cyanobacteria is both scientifically compelling and technologically relevant.

Trace metals, including magnesium (Mg), zinc (Zn), copper (Cu), iron (Fe), and others, are integral to the growth and metabolic processes of cyanobacteria. These metal elements are requisite for numerous enzymatic reactions and are essential for sustaining the physiological integrity of cyanobacterial species. With the addition of metal concentrations, the EPS content in most cyanobacteria significantly increased (Figure 5). The influence of Mg^2+^ on the synthesis of cyanobacterial EPSs exhibits significant genus specificity. Studies have demonstrated an increase in EPS content in *Cyanothece* and *Microcystis aeruginosa* upon the addition of Mg^2+^ [32,65], both cyanobacteria belonging to the family Chroococcaceae. However, a contrasting response was observed in *Nostoc muscorum*, where the addition of Mg^2+^ led to a decrease in EPS content [62]. This suggests that different cyanobacteria may respond variably to Mg^2+^. Additionally, the addition of Zn^2+^ has been reported to significantly increase the EPS yield in *Synechococcus elongatus* PCC 7942 and *Phormidium autumnale* [66,67]. Meanwhile, Loustau et al. [67] found that the addition of Cu^2+^ significantly enhanced the EPS content in *P. autumnale*. The study demonstrated that lead Pb^2+^ significantly inhibited the growth and EPS production in *Arthrospira platensis*, while different strains showed significant differences in tolerance to Pb^2+^ stress [33]. Moreover, the supplementation of Ca^2+^ has been confirmed to enhance the production of cyanobacterial EPSs [22,48,65,68]. These findings reveal that, in addition to trace metal ions, Ca^2+^ may also significantly impact the synthesis and secretion of cyanobacterial EPSs. Despite this, research on the impact of trace metal elements such as iron (Fe) on the synthesis of cyanobacterial EPSs remains relatively scarce. Further exploration of the potential effects of these metal elements is warranted, as they may hold significant implications for the adaptability of cyanobacteria under various environmental conditions and the potential of their EPSs in environmental remediation and biotechnological applications.

Carbon dioxide (CO_2_) is an essential substrate for photosynthesis in cyanobacteria, significantly affecting their growth and polysaccharide production. The CO_2_ concentration critically regulates the biosynthesis and properties of cyanobacterial EPSs, highlighting its role in tailoring polysaccharide output and characteristics [69]. Many studies have reported that increased CO_2_ availability can effectively enhance the production of EPSs in cyanobacteria [11,29,49,54,55,70], with a particular focus on the genus *Nostoc* (Figure 5). These studies suggest that elevated CO_2_ levels stimulate the rate of carbon fixation, leading to an increase in cyanobacterial EPS production [71]. Given the positive effect of CO_2_ on improving cyanobacterial EPS production, future strategies could involve the use of continuous or semi-continuous CO_2_-enriched aeration to maximize EPS content in *Nostoc* species. This approach could be particularly beneficial for optimizing the EPS production in industrial and biotechnological applications.

## 4. Conclusions

This study provides a comprehensive analysis of the effects of various abiotic factors on the production of EPSs in cyanobacteria, highlighting the importance of understanding multi-factor interactions and genus-specific adaptations. Through a systematic review and meta-analysis of published data, we have identified key abiotic factors, including light, temperature, pH, and nutritional conditions, that significantly influence EPS biosynthesis. The research findings indicate that light intensity and quality, as well as nitrogen and phosphorus availability, are the most influential factors affecting EPS production. High light intensities and optimal nutrient conditions generally enhance EPS synthesis. Optimal temperature ranges and pH levels are crucial for maximizing EPS production. Cyanobacteria exhibit genus-specific responses to temperature and pH variations, with some species showing increased EPS production under stress conditions. The addition of specific nutrients, such as NaCl, trace metals (e.g., Mg, Zn, Cu), and elevated CO_2_ levels, significantly impacts EPS production. The response to these factors varies among different cyanobacterial genera, highlighting the need for genus-specific optimization strategies. Different cyanobacterial genera exhibit distinct responses to abiotic factors, emphasizing the importance of tailored cultivation conditions to optimize EPS yields.

Understanding the complex interplay between these abiotic factors and cyanobacterial metabolism is essential for optimizing EPS production. While significant progress has been made in laboratory settings, there is a need for more research on the combined effects of multiple factors and the translation of laboratory findings to field conditions. Future studies should focus on elucidating the molecular mechanisms underlying abiotic regulation of EPS synthesis and exploring the potential of cyanobacterial EPSs in practical applications, such as ecosystem restoration, wastewater treatment, and bioenergy production. This review provides a comprehensive theoretical foundation for optimizing EPS production across diverse cyanobacterial genera and underscores the importance of integrating laboratory and field research to bridge the gap between theoretical knowledge and real-world applications.

## Figures and Tables

**Table 1:** Culture medium for different genera of cyanobacteria.

	Culture_Medium	BG-11	BG-11_0_	Chu-10	Alkaline	Zarrouk	YBCII	F/2	ASN III	Medium-18
*Alage_Genus*										
*Anabaena*		1	0	0	0	0	0	0	0	1
*Cyanothece*		1	0	0	0	0	0	0	0	0
*Gloeocapsa*		1	0	0	0	0	0	0	0	0
*Gloeothece*		1	0	0	0	0	0	0	0	0
*Lyngbya*		1	0	0	0	0	0	0	0	0
*Microcoleus*		1	0	0	0	0	0	0	0	0
*Nostoc*		1	1	0	0	0	0	0	0	0
*Phormidium*		1	1	0	1	0	0	0	0	0
*Scytonema*		1	0	0	0	0	0	0	0	0
*Spirulina*		1	0	1	0	1	0	0	0	0
*Synechocystis*		1	0	0	0	0	0	1	1	0
*Tolypothrix*		1	0	0	0	0	0	0	0	0
*Trichodesmium*		0	0	0	0	0	1	0	0	0

Note: 1 = used; 0 = not used.

## Data Availability

No new data were created or analyzed in this study.

## Supplementary Materials

The following supporting information can be downloaded at: https://www.mdpi.com/article/10.3390/metabo15020131/s1, Table S1: Different blue-green algae culture media; Table S2: Summary table of exopolysaccharide (EPS) production for different cyanobacteria under abiotic culture conditions.
