# Supplementary material for: Meta-Analysis of Abiotic Conditions Affecting Exopolysaccharide Production in Cyanobacteria
Source: Metabolites. 2025 Feb 14;15(2):131. doi: 10.3390/metabo15020131 (PMC11857606; doi:10.3390/metabo15020131)
Supplement: Supplementary file 1 [file metabolites-15-00131-s001.zip › metabolites-3460847-supplementary.pdf]

Table S1 Different blue-green algae culture media

Alage_genus	Alage_species	Growth_condition	Reference
Anabaena	*Gloeocapsa gelatinosa*	Medium-18	33
Anabaena	*Microcystis aeruginosa* FACHB 927	BG-11 medium	40
Anabaena	*Microcystis aeruginosa* FACHB 912	BG-11 medium	42
Anabaena	*Microcystis aeruginosa* FACHB 469	BG-11 medium	42
Cyanothece	*Synechococcus elongatus* PCC 7942	BG-11 medium	65
Cyanothece	*Microcystis aeruginosa* PCC 7820	BG-11 medium	22
Gloeocapsa	*Synechocystis* sp. BASO511	BG-11 medium	62
Gloeothece	*Chroomonas* sp.	BG11 medium	20
Lyngbya	*Nostoc flagelliforme*	BG-11 medium	35, 46, 47
Microcoleus	*Anabaena torulosa*	BG-11 medium	59
Nostoc	*Anabaena* sp. PCC 7120	$BG\text{-}11_0$ medium (BG11 without $NaNO_3$)	69
Nostoc	*Anabaena* sp. ATCC 33047	$BG\text{-}11_0$ medium (BG11 without $NaNO_3$)	29
Nostoc	*Nostoc* PCC 8113	$BG\text{-}11_0$ medium (BG11 without $NaNO_3$)	34
Nostoc	*Nostoc* sp. (FACHB 892)	BG-11 medium	36
Nostoc	*Nostoc* PCC 7936	$BG\text{-}11_0$ medium (BG11 without $NaNO_3$)	49
Nostoc	*Nostoc commune* CANT2 UAM 817	BG-11 medium	23
phormidium	*Nostoc muscorum*	$BG\text{-}11_0$ medium (BG11 without $NaNO_3$)	61
phormidium	*Nostoc* sp. PCC 7120	BG-11 medium	63
phormidium	*Spirulina* sp.	Alkaline	59
Scytonema	*Phormidium* sp.	BG-11 medium	59
Scytonema	*Microcoleus vaginatus*	BG-11 medium	37
Spirulina	*Lyngbya stagnina*	Chu-10 medium	48
Spirulina	*Spirulina platensis*	Zarrouk	30
Spirulina	*Arthrospira platensis*	modified Zarrouk's	31
Spirulina	*Spirulina platensis*	Zarrouk	25
Spirulina	*Arthrospira platensis* C1 (PCC 9438)	Zarrouk	26
Spirulina	*Phormidium autumnale*	BG-11 medium	66
Synechocystis	*Scytonema tolypothrichoides*	BG-11 medium	38
Synechocystis	*Tolypothrix bouteillei*	BG-11 medium	38
Synechocystis	*Cyanothece* sp. 113	F/2 medium	32
Synechocystis	*Cyanothece* ATCC 51142	ASN III medium	56
Tolypothrix	*Cyanobacterium aponinum* PCC 10605	BG-11 medium	39
Microcoleus	*Microcoleus vaginatus* Gom.	BG-11 medium	21
Trichodesmium	*Trichodesmium erythraeum* IMS101	YBCII medium	54, 55
	cyanobacterial strains HSaC	BG-11 medium	24

Table S2 Summary table of exopolysaccharide (EPS) production for different cyanobacteria under various abiotic culture conditions

Reference	Alage_family	Alage_genus	Alage_species	EPS_content	unit	Growth_environment	culture_temperature	Growth_light	Growth_light intensity	Growth_condition	Culture_environment	Culture_environment size	pH
11	Nostoc	*Nostoc*	*Nostoc flagelliforme*	20.97	mg/L DW		25°C	white	60 μmol photons $m^{-2} s^{-1}$	BG-11 medium	shake-flasks	500 mL	
11	Nostoc	*Nostoc*	*Nostoc flagelliforme*	37.03	mg/L DW		25°C	blue light	60 μmol photons $m^{-2} s^{-1}$	BG-11 medium	shake-flasks	500 mL	
11	Nostoc	*Nostoc*	*Nostoc flagelliforme*	27.33	mg/L DW		25°C	red light	60 μmol photons $m^{-2} s^{-1}$	BG-11 medium	shake-flasks	500 mL	
11	Nostoc	*Nostoc*	*Nostoc flagelliforme*	6.98	mg/L DW	0 g/L $NaHCO_3$	25°C	cool-white fluorescent	60 μmol photons $m^{-2} s^{-1}$	BG-11 medium	shake-flasks	500 mL	
11	Nostoc	*Nostoc*	*Nostoc flagelliforme*	28.79	mg/L DW	1.26 g/L $NaHCO_3$	25°C	cool-white fluorescent	60 μmol photons $m^{-2} s^{-1}$	BG-11 medium	shake-flasks	500 mL	
11	Nostoc	*Nostoc*	*Nostoc flagelliforme*	16.06	mg/L DW	2.94 g/L $NaHCO_3$	25°C	cool-white fluorescent	60 μmol photons $m^{-2} s^{-1}$	BG-11 medium	shake-flasks	500 mL	
11	Nostoc	*Nostoc*	*Nostoc flagelliforme*	42.02	mg/L DW	0 g/L $NaNO_3$	25°C	cool-white fluorescent	60 μmol photons $m^{-2} s^{-1}$	BG-11 medium	shake-flasks	500 mL	
11	Nostoc	*Nostoc*	*Nostoc flagelliforme*	4	mg/L DW	NH_4Cl	25°C	cool-white fluorescent	60 μmol photons $m^{-2} s^{-1}$	BG-11 medium	shake-flasks	500 mL	
11	Nostoc	*Nostoc*	*Nostoc flagelliforme*	12	mg/L DW	$CO(NH_2)_2$	25°C	cool-white fluorescent	60 μmol photons $m^{-2} s^{-1}$	BG-11 medium	shake-flasks	500 mL	
11	Nostoc	*Nostoc*	*Nostoc flagelliforme*	32.02	mg/L DW	1.5g/L $NaNO_3$	25°C	cool-white fluorescent	60 μmol photons $m^{-2} s^{-1}$	BG-11 medium	shake-flasks	500 mL	
11	Nostoc	*Nostoc*	*Nostoc flagelliforme*	13.01	mg/L DW	4.5g/L $NaNO_3$	25°C	cool-white fluorescent	60 μmol photons $m^{-2} s^{-1}$	BG-11 medium	shake-flasks	500 mL	
12	Nostoc	*Nostoc*	*Nostoc flagelliforme*	226.05	mg/g DW		25°C	white	60 μmol photons $m^{-2} s^{-1}$	BG-11 medium	shake-flasks	500 mL	
12	Nostoc	*Nostoc*	*Nostoc flagelliforme*	264.38	mg/g DW		25°C	UVB radiation	60 μmol photons $m^{-2} s^{-1}$	BG-11 medium	shake-flasks	500 mL	
12	Nostoc	*Nostoc*	*Nostoc flagelliforme*	377.4	mg/g DW		25°C	red light	60 μmol photons $m^{-2} s^{-1}$	BG-11 medium	shake-flasks	500 mL	
12	Nostoc	*Nostoc*	*Nostoc flagelliforme*	273.95	mg/g DW		25°C	blue light	60 μmol photons $m^{-2} s^{-1}$	BG-11 medium	shake-flasks	500 mL	
12	Nostoc	*Nostoc*	*Nostoc flagelliforme*	285.44	mg/g DW		25°C	mixed light (R:B = 7:15)	60 μmol photons $m^{-2} s^{-1}$	BG-11 medium	shake-flasks	500 mL	
20	Cryptomonadaceae	*chroomonas*	*Chroomonas* sp.	18.78	mg/L	4 mM $NaNO_3$	29°C		156 μmol photons $m^{-2} s^{-1}$	BG11 medium		150 mL	
20	Cryptomonadaceae	*chroomonas*	*Chroomonas* sp.	24.61	mg/L	8 mM $NaNO_3$	29°C		156 μmol photons $m^{-2} s^{-1}$	BG11 medium		150 mL	
20	Cryptomonadaceae	*chroomonas*	*Chroomonas* sp.	28.85	mg/L	12 mM $NaNO_3$	29°C		156 μmol photons $m^{-2} s^{-1}$	BG11 medium		150 mL	
21	Oscillatoriaceae	*Microcoleus*	*Microcoleus vaginitus*	9.8	mg/g DW	0 mmol/L NaCl	25°C		40 μmol photons $m^{-2} s^{-1}$	BG-11 medium			
21	Oscillatoriaceae	*Microcoleus*	*Microcoleus vaginitus*	11.6	mg/g DW	50 mmol/L NaCl	25°C		40 μmol photons $m^{-2} s^{-1}$	BG-11 medium			
21	Oscillatoriaceae	*Microcoleus*	*Microcoleus vaginitus*	13.6	mg/g DW	100 mmol/L NaCl	25°C		40 μmol photons $m^{-2} s^{-1}$	BG-11 medium			
21	Oscillatoriaceae	*Microcoleus*	*Microcoleus vaginitus*	15.4	mg/g DW	300 mmol/L NaCl	25°C		40 μmol photons $m^{-2} s^{-1}$	BG-11 medium			
21	Oscillatoriaceae	*Microcoleus*	*Microcoleus vaginitus*	15.8	mg/g DW	500 mmol/L NaCl	25°C		40 μmol photons $m^{-2} s^{-1}$	BG-11 medium			
21	Oscillatoriaceae	*Microcoleus*	*Microcoleus vaginitus*	14.8	mg/g DW	700 mmol/L NaCl	25°C		40 μmol photons $m^{-2} s^{-1}$	BG-11 medium			
22	Chrococcaceae	*Synechocystis*	*Microcystis aeruginosa* PCC 7820	2.47	pg/cell	20 mg/L $CaCl_2$	20°C		30 μmol photons $m^{-2} s^{-1}$	BG-11 medium		200 mL	7
22	Chrococcaceae	*Synechocystis*	*Microcystis aeruginosa* PCC 7820	3.13	pg/cell	40 mg/L $CaCl_2$	20°C		30 μmol photons $m^{-2} s^{-1}$	BG-11 medium		200 mL	7
22	Chrococcaceae	*Synechocystis*	*Microcystis aeruginosa* PCC 7820	3.42	pg/cell	60 mg/L $CaCl_2$	20°C		30 μmol photons $m^{-2} s^{-1}$	BG-11 medium		200 mL	7
22	Chrococcaceae	*Synechocystis*	*Microcystis aeruginosa* PCC 7820	3.31	pg/cell	80 mg/L $CaCl_2$	20°C		30 μmol photons $m^{-2} s^{-1}$	BG-11 medium		200 mL	7
22	Chrococcaceae	*Synechocystis*	*Microcystis aeruginosa* PCC 7820	3.88	pg/cell	100 mg/L $CaCl_2$	20°C		30 μmol photons $m^{-2} s^{-1}$	BG-11 medium		200 mL	7
23	Nostoc	*Nostoc*	*Nostoc commune* CANT2 UAM 817	1.27114	g/L		25°C		70 μmol photons $m^{-2} s^{-1}$	BG11 medium			
23	Nostoc	*Nostoc*	*Nostoc commune* CANT2 UAM 817	2.17915	g/L		25°C		70 μmol photons $m^{-2} s^{-1}$	BG11o medium (BG11 without $NaNO_3$)			
24			*Cyanobacterial* strains HSaC	0.23	mg/g DW	0 mmol/L NaCl	25°C		50 μmol photons $m^{-2} s^{-1}$	BG-11 medium			
24			*Cyanobacterial* strains HSaC	0.47	mg/g DW	60 mmol/L NaCl	25°C		50 μmol photons $m^{-2} s^{-1}$	BG-11 medium			
24			*Cyanobacterial* strains HSaC	0.71	mg/g DW	120 mmol/L NaCl	25°C		50 μmol photons $m^{-2} s^{-1}$	BG-11 medium			
24			*Cyanobacterial* strains HSaC	0.84	mg/g DW	180 mmol/L NaCl	25°C		50 μmol photons $m^{-2} s^{-1}$	BG-11 medium			
24			*Cyanobacterial* strains HSaC	0.79	mg/g DW	240 mmol/L NaCl	25°C		50 μmol photons $m^{-2} s^{-1}$	BG-11 medium			
24			*Cyanobacterial* strains HSaC	0.64	mg/g DW	300 mmol/L NaCl	25°C		50 μmol photons $m^{-2} s^{-1}$	BG-11 medium			
25	Oscillatoriaceae	*Spirulina*	*Spirulina platensis*	31.05	mg/g DW	45 mg/L $NaNO_3$	25°C		9 μmol photons $m^{-2} s^{-1}$	Zarrouk		3000mL	10.5
25	Oscillatoriaceae	*Spirulina*	*Spirulina platensis*	28.09	mg/g DW	128 mg/L $NaNO_3$	25°C		9 μmol photons $m^{-2} s^{-1}$	Zarrouk		3000mL	10.5
25	Oscillatoriaceae	*Spirulina*	*Spirulina platensis*	23.62	mg/g DW	293 mg/L $NaNO_3$	25°C		9 μmol photons $m^{-2} s^{-1}$	Zarrouk		3000mL	10.5
25	Oscillatoriaceae	*Spirulina*	*Spirulina platensis*	25.26	mg/g DW	412 mg/L $NaNO_3$	25°C		9 μmol photons $m^{-2} s^{-1}$	Zarrouk		3000mL	10.5
26	Oscillatoriaceae	*Spirulina*	*Arthrospira platensis* C1	150.24	mg/g cell		35°C		100 μmol photons $m^{-2} s^{-1}$	Zarrouk			
26	Oscillatoriaceae	*Spirulina*	*Arthrospira platensis* C1	215.2	mg/g cell		35°C		100 μmol photons $m^{-2} s^{-1}$	Zarrouk without N			
26	Oscillatoriaceae	*Spirulina*	*Arthrospira platensis* C1	131.26	mg/g cell		42°C		100 μmol photons $m^{-2} s^{-1}$	Zarrouk without N			
26	Oscillatoriaceae	*Spirulina*	*Arthrospira* sp. PCC 8005	34.99	mg/L		30°C		43 μmol photons $m^{-2} s^{-1}$	Zarrouk			
26	Oscillatoriaceae	*Spirulina*	*Arthrospira* sp. PCC 8005	88.23	mg/L		30°C		43 μmol photons $m^{-2} s^{-1}$	Zarrouk without N			
29	Nostoc	*Anabaena*	*Anabaena* sp. ATCC 33047	17200	mg/L	N_2 (air)	40°C	cool-white fluorescent	460 μmol photons $m^{-2} s^{-1}$	BG11o medium (BG11 without $NaNO_3$)	photobioreactor		8
29	Nostoc	*Anabaena*	*Anabaena* sp. ATCC 33047	2000	mg/L	KNO_3 (20 mM)	40°C	cool-white fluorescent	460 μmol photons $m^{-2} s^{-1}$	BG11o medium (BG11 without $NaNO_3$)	photobioreactor		8
29	Nostoc	*Anabaena*	*Anabaena* sp. ATCC 33047	2700	mg/L	NH_4Cl (5 mM)	40°C	cool-white fluorescent	460 μmol photons $m^{-2} s^{-1}$	BG11o medium (BG11 without $NaNO_3$)	photobioreactor		8
29	Nostoc	*Anabaena*	*Anabaena* sp. ATCC 33047	13500	mg/L	0 mM NaCl	40°C	cool-white fluorescent	460 μmol photons $m^{-2} s^{-1}$	BG11o medium (BG11 without $NaNO_3$)	photobioreactor		8
29	Nostoc	*Anabaena*	*Anabaena* sp. ATCC 33047	9200	mg/L	85 mM NaCl	40°C	cool-white fluorescent	460 μmol photons $m^{-2} s^{-1}$	BG11o medium (BG11 without $NaNO_3$)	photobioreactor		8
29	Nostoc	*Anabaena*	*Anabaena* sp. ATCC 33047	7900	mg/L	170 mM NaCl	40°C	cool-white fluorescent	460 μmol photons $m^{-2} s^{-1}$	BG11o medium (BG11 without $NaNO_3$)	photobioreactor		8
29	Nostoc	*Anabaena*	*Anabaena* sp. ATCC 33047	3900	mg/L	340 mM NaCl	40°C	cool-white fluorescent	460 μmol photons $m^{-2} s^{-1}$	BG11o medium (BG11 without $NaNO_3$)	photobioreactor		8
29	Nostoc	*Anabaena*	*Anabaena* sp. ATCC 33047	3500	mg/L	425 mM NaCl	40°C	cool-white fluorescent	460 μmol photons $m^{-2} s^{-1}$	BG11o medium (BG11 without $NaNO_3$)	photobioreactor		8
29	Nostoc	*Anabaena*	*Anabaena* sp. ATCC 33047	3900	mg/L	510 mM NaCl	40°C	cool-white fluorescent	460 μmol photons $m^{-2} s^{-1}$	BG11o medium (BG11 without $NaNO_3$)	photobioreactor		8
29	Nostoc	*Anabaena*	*Anabaena* sp. ATCC 33047	11759.3	mg/L		40°C	cool-white fluorescent	460 μmol photons $m^{-2} s^{-1}$	BG11o medium (BG11 without $NaNO_3$)	photobioreactor		8
29	Nostoc	*Anabaena*	*Anabaena* sp. ATCC 33047	3098.23	mg/L		40°C	cool-white fluorescent	185 μmol photons $m^{-2} s^{-1}$	BG11o medium (BG11 without $NaNO_3$)	photobioreactor		8
29	Nostoc	*Anabaena*	*Anabaena* sp. ATCC 33047	2514.2	mg/L		40°C	cool-white fluorescent	345 μmol photons $m^{-2} s^{-1}$	BG11o medium (BG11 without $NaNO_3$)	photobioreactor		8
29	Nostoc	*Anabaena*	*Anabaena* sp. ATCC 33047	11500	mg/L		40°C	cool-white fluorescent	920 μmol photons $m^{-2} s^{-1}$	BG11o medium (BG11 without $NaNO_3$)	photobioreactor		8
29	Nostoc	*Anabaena*	*Anabaena* sp. ATCC 33047	10634.7	mg/L		40°C	cool-white fluorescent	1840 μmol photons $m^{-2} s^{-1}$	BG11o medium (BG11 without $NaNO_3$)	photobioreactor		8
29	Nostoc	*Anabaena*	*Anabaena* sp. ATCC 33047	4100	mg/L	6% CO_2, 50 L/h	40°C	cool-white fluorescent	460 μmol photons $m^{-2} s^{-1}$	BG11o medium (BG11 without $NaNO_3$)	photobioreactor		8
29	Nostoc	*Anabaena*	*Anabaena* sp. ATCC 33047	4900	mg/L	6% CO_2, 150 L/h	40°C	cool-white fluorescent	460 μmol photons $m^{-2} s^{-1}$	BG11o medium (BG11 without $NaNO_3$)	photobioreactor		8
29	Nostoc	*Anabaena*	*Anabaena* sp. ATCC 33047	14600	mg/L	6% CO_2, 250 L/h	40°C	cool-white fluorescent	460 μmol photons $m^{-2} s^{-1}$	BG11o medium (BG11 without $NaNO_3$)	photobioreactor		8
30	Oscillatoriaceae	*Spirulina*	*Spirulina platensis*	67.69	mg/g DW		23°C	cool-white fluorescent	24 μmol photons $m^{-2} s^{-1}$	Zarrouk	tank	150L	9.4-9.9
30	Oscillatoriaceae	*Spirulina*	*Spirulina platensis*	105.22	mg/g DW		23°C	cool-white fluorescent	96 μmol photons $m^{-2} s^{-1}$	Zarrouk	tank	150L	9.4-9.9
30	Oscillatoriaceae	*Spirulina*	*Spirulina platensis*	146.45	mg/g DW		23°C	cool-white fluorescent	192 μmol photons $m^{-2} s^{-1}$	Zarrouk	tank	150L	9.4-9.9
30	Oscillatoriaceae	*Spirulina*	*Spirulina platensis*	54.59	mg/g DW		18 °C	cool-white fluorescent	36 μmol photons $m^{-2} s^{-1}$	Zarrouk	tank	150L	9.4-9.9
30	Oscillatoriaceae	*Spirulina*	*Spirulina platensis*	69.58	mg/g DW		23 °C	cool-white fluorescent	36 μmol photons $m^{-2} s^{-1}$	Zarrouk	tank	150L	9.4-9.9
30	Oscillatoriaceae	*Spirulina*	*Spirulina platensis*	79.79	mg/g DW		28 °C	cool-white fluorescent	36 μmol photons $m^{-2} s^{-1}$	Zarrouk	tank	150L	9.4-9.9
30	Oscillatoriaceae	*Spirulina*	*Spirulina platensis*	96.96	mg/g DW		33 °C	cool-white fluorescent	36 μmol photons $m^{-2} s^{-1}$	Zarrouk	tank	150L	9.4-9.9
30	Oscillatoriaceae	*Spirulina*	*Spirulina platensis*	141.06	mg/g DW		38 °C	cool-white fluorescent	36 μmol photons $m^{-2} s^{-1}$	Zarrouk	tank	150L	9.4-9.9
30	Oscillatoriaceae	*Spirulina*	*Spirulina platensis*	41.03	mg/g DW	0 mol/L NaCl	28 °C	cool-white fluorescent	96 μmol photons $m^{-2} s^{-1}$	Zarrouk	tank	150L	9.4-9.9
30	Oscillatoriaceae	*Spirulina*	*Spirulina platensis*	53.18	mg/g DW	0 mol/L NaCl	28 °C	cool-white fluorescent	96 μmol photons $m^{-2} s^{-1}$	Zarrouk	tank	150L	9.4-9.9
30	Oscillatoriaceae	*Spirulina*	*Spirulina platensis*	70.09	mg/g DW	0 mol/L NaCl	28 °C	cool-white fluorescent	96 μmol photons $m^{-2} s^{-1}$	Zarrouk	tank	150L	9.4-9.9
30	Oscillatoriaceae	*Spirulina*	*Spirulina platensis*	89.63	mg/g DW	0 mol/L NaCl	28 °C	cool-white fluorescent	96 μmol photons $m^{-2} s^{-1}$	Zarrouk	tank	150L	9.4-9.9
30	Oscillatoriaceae	*Spirulina*	*Spirulina platensis*	91.62	mg/g DW	0.25 mol/L NaCl	28 °C	cool-white fluorescent	96 μmol photons $m^{-2} s^{-1}$	Zarrouk	tank	150L	9.4-9.9
30	Oscillatoriaceae	*Spirulina*	*Spirulina platensis*	142.84	mg/g DW	0.25 mol/L NaCl	28 °C	cool-white fluorescent	96 μmol photons $m^{-2} s^{-1}$	Zarrouk	tank	150L	9.4-9.9
30	Oscillatoriaceae	*Spirulina*	*Spirulina platensis*	138.54	mg/g DW	0.25 mol/L NaCl	28 °C	cool-white fluorescent	96 μmol photons $m^{-2} s^{-1}$	Zarrouk	tank	150L	9.4-9.9
30	Oscillatoriaceae	*Spirulina*	*Spirulina platensis*	127.85	mg/g DW	0.25 mol/L NaCl	28 °C	cool-white fluorescent	96 μmol photons $m^{-2} s^{-1}$	Zarrouk	tank	150L	9.4-9.9
30	Oscillatoriaceae	*Spirulina*	*Spirulina platensis*	125.24	mg/g DW	0.5 mol/L NaCl	28 °C	cool-white fluorescent	96 μmol photons $m^{-2} s^{-1}$	Zarrouk	tank	150L	9.4-9.9
30	Oscillatoriaceae	*Spirulina*	*Spirulina platensis*	174.35	mg/g DW	0.5 mol/L NaCl	28 °C	cool-white fluorescent	96 μmol photons $m^{-2} s^{-1}$	Zarrouk	tank	150L	9.4-9.9
30	Oscillatoriaceae	*Spirulina*	*Spirulina platensis*	159.72	mg/g DW	0.5 mol/L NaCl	28 °C	cool-white fluorescent	96 μmol photons $m^{-2} s^{-1}$	Zarrouk	tank	150L	9.4-9.9
30	Oscillatoriaceae	*Spirulina*	*Spirulina platensis*	142.42	mg/g DW	0.5 mol/L NaCl	28 °C	cool-white fluorescent	96 μmol photons $m^{-2} s^{-1}$	Zarrouk	tank	150L	9.4-9.9
30	Oscillatoriaceae	*Spirulina*	*Spirulina platensis*	143.64	mg/g DW	0.75 mol/L NaCl	28 °C	cool-white fluorescent	96 μmol photons $m^{-2} s^{-1}$	Zarrouk	tank	150L	9.4-9.9
30	Oscillatoriaceae	*Spirulina*	*Spirulina platensis*	196.53	mg/g DW	0.75 mol/L NaCl	28 °C	cool-white fluorescent	96 μmol photons $m^{-2} s^{-1}$	Zarrouk	tank	150L	9.4-9.9
30	Oscillatoriaceae	*Spirulina*	*Spirulina platensis*	170.49	mg/g DW	0.75 mol/L NaCl	28 °C	cool-white fluorescent	96 μmol photons $m^{-2} s^{-1}$	Zarrouk	tank	150L	9.4-9.9
30	Oscillatoriaceae	*Spirulina*	*Spirulina platensis*	158.17	mg/g DW	0.75 mol/L NaCl	28 °C	cool-white fluorescent	96 μmol photons $m^{-2} s^{-1}$	Zarrouk	tank	150L	9.4-9.9
30	Oscillatoriaceae	*Spirulina*	*Spirulina platensis*	120.54	mg/g DW	1 mol/L NaCl	28 °C	cool-white fluorescent	96 μmol photons $m^{-2} s^{-1}$	Zarrouk	tank	150L	9.4-9.9
30	Oscillatoriaceae	*Spirulina*	*Spirulina platensis*	146.27	mg/g DW	1 mol/L NaCl	28 °C	cool-white fluorescent	96 μmol photons $m^{-2} s^{-1}$	Zarrouk	tank	150L	9.4-9.9
30	Oscillatoriaceae	*Spirulina*	*Spirulina platensis*	119.78	mg/g DW	1 mol/L NaCl	28 °C	cool-white fluorescent	96 μmol photons $m^{-2} s^{-1}$	Zarrouk	tank	150L	9.4-9.9
30	Oscillatoriaceae	*Spirulina*	*Spirulina platensis*	101.25	mg/g DW	1 mol/L NaCl	28 °C	cool-white fluorescent	96 μmol photons $m^{-2} s^{-1}$	Zarrouk	tank	150L	9.4-9.9
31	Oscillatoriaceae	*Spirulina*	*Arthrospira platensis*	23		0.25 mg/L$NaNO_3$	23°C	cool-white fluorescent	200 μmol photons $m^{-2} s^{-1}$	modified Zarrouk's		300mL	
31	Oscillatoriaceae	*Spirulina*	*Arthrospira platensis*	23.5		1.125 mg/L$NaNO_3$	23°C	cool-white fluorescent	200 μmol photons $m^{-2} s^{-1}$	modified Zarrouk's		300mL	
31	Oscillatoriaceae	*Spirulina*	*Arthrospira platensis*	24.43		2 mg/L$NaNO_3$	23°C	cool-white fluorescent	200 μmol photons $m^{-2} s^{-1}$	modified Zarrouk's		300mL	
31	Oscillatoriaceae	*Spirulina*	*Arthrospira platensis*	27.43		0.25 mg/L$NaNO_3$	23°C	cool-white fluorescent	600 μmol photons $m^{-2} s^{-1}$	modified Zarrouk's		300mL	
31	Oscillatoriaceae	*Spirulina*	*Arthrospira platensis*	39.5		1.125 mg/L$NaNO_3$	23°C	cool-white fluorescent	600 μmol photons $m^{-2} s^{-1}$	modified Zarrouk's		300mL	
31	Oscillatoriaceae	*Spirulina*	*Arthrospira platensis*	17.53		2 mg/L$NaNO_3$	23°C	cool-white fluorescent	600 μmol photons $m^{-2} s^{-1}$	modified Zarrouk's		300mL	
31	Oscillatoriaceae	*Spirulina*	*Arthrospira platensis*	23.67		0.25 mg/L$NaNO_3$	23°C	cool-white fluorescent	1000 μmol photons $m^{-2} s^{-1}$	modified Zarrouk's		300mL	
31	Oscillatoriaceae	*Spirulina*	*Arthrospira platensis*	34		1.125 mg/L$NaNO_3$	23°C	cool-white fluorescent	1000 μmol photons $m^{-2} s^{-1}$	modified Zarrouk's		300mL	
31	Oscillatoriaceae	*Spirulina*	*Arthrospira platensis*	38.73		2 mg/L$NaNO_3$	23°C	cool-white fluorescent	1000 μmol photons $m^{-2} s^{-1}$	modified Zarrouk's		300mL	
32	Cyanobiaceae	*Cyanothece*	*Cyanothece* sp. 113	2850	mg/L	0 mol/L NaCl	29°C	cool-white fluorescent	86 μmol photons $m^{-2} s^{-1}$	F/2 medium	shake-flasks	50mL	
32	Cyanobiaceae	*Cyanothece*	*Cyanothece* sp. 113	4550	mg/L	0.34 mol/L NaCl	29°C	cool-white fluorescent	86 μmol photons $m^{-2} s^{-1}$	F/2 medium	shake-flasks	50mL	
32	Cyanobiaceae	*Cyanothece*	*Cyanothece* sp. 113	5020	mg/L	0.51 mol/L NaCl	29°C	cool-white fluorescent	86 μmol photons $m^{-2} s^{-1}$	F/2 medium	shake-flasks	50mL	
32	Cyanobiaceae	*Cyanothece*	*Cyanothece* sp. 113	5610	mg/L	0.68 mol/L NaCl	29°C	cool-white fluorescent	86 μmol photons $m^{-2} s^{-1}$	F/2 medium	shake-flasks	50mL	
32	Cyanobiaceae	*Cyanothece*	*Cyanothece* sp. 113	5580	mg/L	1.03 mol/L NaCl	29°C	cool-white fluorescent	86 μmol photons $m^{-2} s^{-1}$	F/2 medium	shake-flasks	50mL	
32	Cyanobiaceae	*Cyanothece*	*Cyanothece* sp. 113	4610	mg/L	1.2 mol/L NaCl	29°C	cool-white fluorescent	86 μmol photons $m^{-2} s^{-1}$	F/2 medium	shake-flasks	50mL	
32	Cyanobiaceae	*Cyanothece*	*Cyanothece* sp. 113	4080	mg/L	1.37 mol/L NaCl	29°C	cool-white fluorescent	86 μmol photons $m^{-2} s^{-1}$	F/2 medium	shake-flasks	50mL	
32	Cyanobiaceae	*Cyanothece*	*Cyanothece* sp. 113	3420	mg/L	1.55 mol/L NaCl	29°C	cool-white fluorescent	86 μmol photons $m^{-2} s^{-1}$	F/2 medium	shake-flasks	50mL	
32	Cyanobiaceae	*Cyanothece*	*Cyanothece* sp. 113	3260	mg/L	1.72 mol/L NaCl	29°C	cool-white fluorescent	86 μmol photons $m^{-2} s^{-1}$	F/2 medium	shake-flasks	50mL	
32	Cyanobiaceae	*Cyanothece*	*Cyanothece* sp. 113	7003.19	mg/L	0 mg/L $NaNO_3$	29°C	cool-white fluorescent	86 μmol photons $m^{-2} s^{-1}$	F/2 medium	shake-flasks	50mL	
32	Cyanobiaceae	*Cyanothece*	*Cyanothece* sp. 113	5551.23	mg/L	75 mg/L $NaNO_3$	29°C	cool-white fluorescent	86 μmol photons $m^{-2} s^{-1}$	F/2 medium	shake-flasks	50mL	
32	Cyanobiaceae	*Cyanothece*	*Cyanothece* sp. 113	4863.94	mg/L	120 mg/L $NaNO_3$	29°C	cool-white fluorescent	86 μmol photons $m^{-2} s^{-1}$	F/2 medium	shake-flasks	50mL	
32	Cyanobiaceae	*Cyanothece*	*Cyanothece* sp. 113	4866.42	mg/L	160 mg/L $NaNO_3$	29°C	cool-white fluorescent	86 μmol photons $m^{-2} s^{-1}$	F/2 medium	shake-flasks	50mL	
32	Cyanobiaceae	*Cyanothece*	*Cyanothece* sp. 113	4843.34	mg/L	200 mg/L $NaNO_3$	29°C	cool-white fluorescent	86 μmol photons $m^{-2} s^{-1}$	F/2 medium	shake-flasks	50mL	
32	Cyanobiaceae	*Cyanothece*	*Cyanothece* sp. 113	6044.44	mg/L	0 mg/L NaH_2PO_4	29°C	cool-white fluorescent	86 μmol photons $m^{-2} s^{-1}$	F/2 medium	shake-flasks	50mL	
32	Cyanobiaceae	*Cyanothece*	*Cyanothece* sp. 113	5765.08	mg/L	10 mg/L NaH_2PO_4	29°C	cool-white fluorescent	86 μmol photons $m^{-2} s^{-1}$	F/2 medium	shake-flasks	50mL	
32	Cyanobiaceae	*Cyanothece*	*Cyanothece* sp. 113	5688.89	mg/L	40 mg/L NaH_2PO_4	29°C	cool-white fluorescent	86 μmol photons $m^{-2} s^{-1}$	F/2 medium	shake-flasks	50mL	
32	Cyanobiaceae	*Cyanothece*	*Cyanothece* sp. 113	5790.48	mg/L	60 mg/L NaH_2PO_4	29°C	cool-white fluorescent	86 μmol photons $m^{-2} s^{-1}$	F/2 medium	shake-flasks	50mL	
32	Cyanobiaceae	*Cyanothece*	*Cyanothece* sp. 113	5942.86	mg/L	90 mg/L NaH_2PO_4	29°C	cool-white fluorescent	86 μmol photons $m^{-2} s^{-1}$	F/2 medium	shake-flasks	50mL	
32	Cyanobiaceae	*Cyanothece*	*Cyanothece* sp. 113	5993.65	mg/L	120 mg/L NaH_2PO_4	29°C	cool-white fluorescent	86 μmol photons $m^{-2} s^{-1}$	F/2 medium	shake-flasks	50mL	
32	Cyanobiaceae	*Cyanothece*	*Cyanothece* sp. 113	5993.65	mg/L	160 mg/L NaH_2PO_4	29°C	cool-white fluorescent	86 μmol photons $m^{-2} s^{-1}$	F/2 medium	shake-flasks	50mL	
32	Cyanobiaceae	*Cyanothece*	*Cyanothece* sp. 113	6069.84	mg/L	180 mg/L NaH_2PO_4	29°C	cool-white fluorescent	86 μmol photons $m^{-2} s^{-1}$	F/2 medium	shake-flasks	50mL	
32	Cyanobiaceae	*Cyanothece*	*Cyanothece* sp. 113	6095.24	mg/L	200 mg/L NaH_2PO_4	29°C	cool-white fluorescent	86 μmol photons $m^{-2} s^{-1}$	F/2 medium	shake-flasks	50mL	
32	Cyanobiaceae	*Cyanothece*	*Cyanothece* sp. 113	5652.87	mg/L	0 g/L $MgSO_4$	29°C	cool-white fluorescent	86 μmol photons $m^{-2} s^{-1}$	F/2 medium	shake-flasks	50mL	
32	Cyanobiaceae	*Cyanothece*	*Cyanothece* sp. 113	8121.02	mg/L	0.1 g/L $MgSO_4$	29°C	cool-white fluorescent	86 μmol photons $m^{-2} s^{-1}$	F/2 medium	shake-flasks	50mL	
32	Cyanobiaceae	*Cyanothece*	*Cyanothece* sp. 113	10429.9	mg/L	0.2 g/L $MgSO_4$	29°C	cool-white fluorescent	86 μmol photons $m^{-2} s^{-1}$	F/2 medium	shake-flasks	50mL	
32	Cyanobiaceae	*Cyanothece*	*Cyanothece* sp. 113	11783.4	mg/L	0.3 g/L $MgSO_4$	29°C	cool-white fluorescent	86 μmol photons $m^{-2} s^{-1}$	F/2 medium	shake-flasks	50mL	
32	Cyanobiaceae	*Cyanothece*	*Cyanothece* sp. 113	13535	mg/L	0.4 g/L $MgSO_4$	29°C	cool-white fluorescent	86 μmol photons $m^{-2} s^{-1}$	F/2 medium	shake-flasks	50mL	
32	Cyanobiaceae	*Cyanothece*	*Cyanothece* sp. 113	14570.1	mg/L	0.5 g/L $MgSO_4$	29°C	cool-white fluorescent	86 μmol photons $m^{-2} s^{-1}$	F/2 medium	shake-flasks	50mL	
32	Cyanobiaceae	*Cyanothece*	*Cyanothece* sp. 113	15127.4	mg/L	0.6 g/L $MgSO_4$	29°C	cool-white fluorescent	86 μmol photons $m^{-2} s^{-1}$	F/2 medium	shake-flasks	50mL	
32	Cyanobiaceae	*Cyanothece*	*Cyanothece* sp. 113	16719.7	mg/L	0.7 g/L $MgSO_4$	29°C	cool-white fluorescent	86 μmol photons $m^{-2} s^{-1}$	F/2 medium	shake-flasks	50mL	
32	Cyanobiaceae	*Cyanothece*	*Cyanothece* sp. 113	17754.8	mg/L	0.8 g/L $MgSO_4$	29°C	cool-white fluorescent	86 μmol photons $m^{-2} s^{-1}$	F/2 medium	shake-flasks	50mL	
32	Cyanobiaceae	*Cyanothece*	*Cyanothece* sp. 113	19347.1	mg/L	0.9 g/L $MgSO_4$	29°C	cool-white fluorescent	86 μmol photons $m^{-2} s^{-1}$	F/2 medium	shake-flasks	50mL	
32	Cyanobiaceae	*Cyanothece*	*Cyanothece* sp. 113	17117.8	mg/L	1.0 g/L $MgSO_4$	29°C	cool-white fluorescent	86 μmol photons $m^{-2} s^{-1}$	F/2 medium	shake-flasks	50mL	
32	Cyanobiaceae	*Cyanothece*	*Cyanothece* sp. 113	13857.5	mg/L	1.1 g/L $MgSO_4$	29°C	cool-white fluorescent	86 μmol photons $m^{-2} s^{-1}$	F/2 medium	shake-flasks	50mL	
32	Cyanobiaceae	*Cyanothece*	*Cyanothece* sp. 113	3386.19	mg/L		25°C	cool-white fluorescent	86 μmol photons $m^{-2} s^{-1}$	F/2 medium	shake-flasks	50mL	
32	Cyanobiaceae	*Cyanothece*	*Cyanothece* sp. 113	9403.64	mg/L		27°C	cool-white fluorescent	86 μmol photons $m^{-2} s^{-1}$	F/2 medium	shake-flasks	50mL	
32	Cyanobiaceae	*Cyanothece*	*Cyanothece* sp. 113	18296.1	mg/L		29°C	cool-white fluorescent	86 μmol photons $m^{-2} s^{-1}$	F/2 medium	shake-flasks	50mL	
32	Cyanobiaceae	*Cyanothece*	*Cyanothece* sp. 113	18306.7	mg/L		31°C	cool-white fluorescent	86 μmol photons $m^{-2} s^{-1}$	F/2 medium	shake-flasks	50mL	
32	Cyanobiaceae	*Cyanothece*	*Cyanothece* sp. 113	7901.89	mg/L		33°C	cool-white fluorescent	86 μmol photons $m^{-2} s^{-1}$	F/2 medium	shake-flasks	50mL	
32	Cyanobiaceae	*Cyanothece*	*Cyanothece* sp. 113	5995.69	mg/L		35°C	cool-white fluorescent	86 μmol photons $m^{-2} s^{-1}$	F/2 medium	shake-flasks	50mL	
32	Cyanobiaceae	*Cyanothece*	*Cyanothece* sp. 113	3724.38	mg/L			cool-white fluorescent	47 μmol photons $m^{-2} s^{-1}$	F/2 medium	shake-flasks	50mL	
32	Cyanobiaceae	*Cyanothece*	*Cyanothece* sp. 113	7128.76	mg/L			cool-white fluorescent	58 μmol photons $m^{-2} s^{-1}$	F/2 medium	shake-flasks	50mL	
32	Cyanobiaceae	*Cyanothece*	*Cyanothece* sp. 113	18560.3	mg/L			cool-white fluorescent	80 μmol photons $m^{-2} s^{-1}$	F/2 medium	shake-flasks	50mL	
32	Cyanobiaceae	*Cyanothece*	*Cyanothece* sp. 113	17171.3	mg/L			cool-white fluorescent	100 μmol photons $m^{-2} s^{-1}$	F/2 medium	shake-flasks	50mL	
32	Cyanobiaceae	*Cyanothece*	*Cyanothece* sp. 113	15723.9	mg/L			cool-white fluorescent	110 μmol photons $m^{-2} s^{-1}$	F/2 medium	shake-flasks	50mL	
32	Cyanobiaceae	*Cyanothece*	*Cyanothece* sp. 113	11883.3	mg/L			cool-white fluorescent	120 μmol photons $m^{-2} s^{-1}$	F/2 medium	shake-flasks	50mL	
32	Cyanobiaceae	*Cyanothece*	*Cyanothece* sp. 113	9486.66	mg/L			cool-white fluorescent	130 μmol photons $m^{-2} s^{-1}$	F/2 medium	shake-flasks	50mL	
32	Cyanobiaceae	*Cyanothece*	*Cyanothece* sp. 113	9048.9	mg/L			cool-white fluorescent	150 μmol photons $m^{-2} s^{-1}$	F/2 medium	shake-flasks	50mL	
32	Cyanobiaceae	*Cyanothece*	*Cyanothece* sp. 113	7031.3	mg/L			cool-white fluorescent	160 μmol photons $m^{-2} s^{-1}$	F/2 medium	shake-flasks	50mL	
33	Chrococcaceae	*Gloeocapsa*	*Gloeocapsa gelatinosa*	19.5	mg/L		25°C	cool-white fluorescent	200 μmol photons $m^{-2} s^{-1}$	Medium-18			7
33	Chrococcaceae	*Gloeocapsa*	*Gloeocapsa gelatinosa*	33	mg/L		25°C	cool-white fluorescent	400 μmol photons $m^{-2} s^{-1}$	Medium-18			7
33	Chrococcaceae	*Gloeocapsa*	*Gloeocapsa gelatinosa*	20	mg/L		25°C	UVB radiation	400 μmol photons $m^{-2} s^{-1}$	Medium-18			7
33	Chrococcaceae	*Gloeocapsa*	*Gloeocapsa gelatinosa*	31.8	mg/L		25°C	cool-white fluorescent	400 μmol photons $m^{-2} s^{-1}$	Medium-18			4
33	Chrococcaceae	*Gloeocapsa*	*Gloeocapsa gelatinosa*	27.8	mg/L		25°C	cool-white fluorescent	400 μmol photons $m^{-2} s^{-1}$	Medium-18 without N			7
34	Nostoc	*Nostoc*	*Nostoc* PCC 8113	455.15	mg/L		30°C	cool-white fluorescent	70 μmol photons $m^{-2} s^{-1}$	BG11o medium (BG11 without $NaNO_3$)	shake-flasks	200 mL	7
34	Nostoc	*Nostoc*	*Nostoc* PCC 8113	574.75	mg/L		30°C	cool-white fluorescent	160 μmol photons $m^{-2} s^{-1}$	BG11o medium (BG11 without $NaNO_3$)	shake-flasks	200 mL	7
34	Nostoc	*Nostoc*	*Nostoc* PCC 7936	664.36	mg/L		30°C	cool-white fluorescent	70 μmol photons $m^{-2} s^{-1}$	BG11o medium (BG11 without $NaNO_3$)	shake-flasks	200 mL	7
34	Nostoc	*Nostoc*	*Nostoc* PCC 7936	864.57	mg/L		30°C	cool-white fluorescent	160 μmol photons $m^{-2} s^{-1}$	BG11o medium (BG11 without $NaNO_3$)	shake-flasks	200 mL	7
34	Nostoc	*Nostoc*	*Nostoc* PCC 7413	1294.4	mg/L		30°C	cool-white fluorescent	70 μmol photons $m^{-2} s^{-1}$	BG11o medium (BG11 without $NaNO_3$)	shake-flasks	200 mL	7
34	Nostoc	*Nostoc*	*Nostoc* PCC 7413	1762.94	mg/L		30°C	cool-white fluorescent	160 μmol photons $m^{-2} s^{-1}$	BG11o medium (BG11 without $NaNO_3$)	shake-flasks	200 mL	7
35	Nostoc	*Nostoc*	*Nostoc flagelliforme*	30.95	mg/L		25°C	red:blue=1:1	20 μmol photons $m^{-2} s^{-1}$	BG-11 medium	shake-flasks	200 mL	7
35	Nostoc	*Nostoc*	*Nostoc flagelliforme*	24.51	mg/L		25°C	red:blue=1:1	40 μmol photons $m^{-2} s^{-1}$	BG-11 medium	shake-flasks	200 mL	7
35	Nostoc	*Nostoc*	*Nostoc flagelliforme*	23.25	mg/L		25°C	red:blue=1:1	60 μmol photons $m^{-2} s^{-1}$	BG-11 medium	shake-flasks	200 mL	7
35	Nostoc	*Nostoc*	*Nostoc flagelliforme*	36.85	mg/L		25°C	red:blue=7:15	20 μmol photons $m^{-2} s^{-1}$	BG-11 medium	shake-flasks	200 mL	7
35	Nostoc	*Nostoc*	*Nostoc flagelliforme*	32.6	mg/L		25°C	red:blue=7:15	40 μmol photons $m^{-2} s^{-1}$	BG-11 medium	shake-flasks	200 mL	7
35	Nostoc	*Nostoc*	*Nostoc flagelliforme*	31	mg/L		25°C	red:blue=7:15	60 μmol photons $m^{-2} s^{-1}$	BG-11 medium	shake-flasks	200 mL	7
35	Nostoc	*Nostoc*	*Nostoc flagelliforme*	46.54	mg/L		25°C	red:blue=15:7	20 μmol photons $m^{-2} s^{-1}$	BG-11 medium	shake-flasks	200 mL	7
35	Nostoc	*Nostoc*	*Nostoc flagelliforme*	24.24	mg/L		25°C	red:blue=15:7	40 μmol photons $m^{-2} s^{-1}$	BG-11 medium	shake-flasks	200 mL	7
35	Nostoc	*Nostoc*	*Nostoc flagelliforme*	24.54	mg/L		25°C	red:blue=15:7	60 μmol photons $m^{-2} s^{-1}$	BG-11 medium	shake-flasks	200 mL	7
35	Nostoc	*Nostoc*	*Nostoc flagelliforme*	46.38	mg/L		25°C	red:blue:green=12:5:5	20 μmol photons $m^{-2} s^{-1}$	BG-11 medium	shake-flasks	200 mL	7
35	Nostoc	*Nostoc*	*Nostoc flagelliforme*	37.83	mg/L		25°C	red:blue:green=12:5:5	40 μmol photons $m^{-2} s^{-1}$	BG-11 medium	shake-flasks	200 mL	7
35	Nostoc	*Nostoc*	*Nostoc flagelliforme*	42.1	mg/L		25°C	red:blue:green=12:5:5	60 μmol photons $m^{-2} s^{-1}$	BG-11 medium	shake-flasks	200 mL	7
35	Nostoc	*Nostoc*	*Nostoc flagelliforme*	27.71	mg/L		25°C	red:blue:green=12:5:2	20 μmol photons $m^{-2} s^{-1}$	BG-11 medium	shake-flasks	200 mL	7
35	Nostoc	*Nostoc*	*Nostoc flagelliforme*	35	mg/L		25°C	red:blue:green=12:5:2	40 μmol photons $m^{-2} s^{-1}$	BG-11 medium	shake-flasks	200 mL	7
35	Nostoc	*Nostoc*	*Nostoc flagelliforme*	36.25	mg/L		25°C	red:blue:green=12:5:2	60 μmol photons $m^{-2} s^{-1}$	BG-11 medium	shake-flasks	200 mL	7
36	Nostoc	*Nostoc*	*Nostoc* sp. (FACHB 892)	56.4	mg/g DW		25°C	cool-white fluorescent	40 μmol photons $m^{-2} s^{-1}$	BG-11 medium	shake-flasks	200 mL	7
36	Nostoc	*Nostoc*	*Nostoc* sp. (FACHB 892)	66.6	mg/g DW		25°C	cool-white fluorescent	80 μmol photons $m^{-2} s^{-1}$	BG-11 medium	shake-flasks	200 mL	7
37	Oscillatoriaceae	*phormidium*	*Microcoleus vaginatus*	94.15	mg/g DW		25°C	cool-white fluorescent	4 μmol photons $m^{-2} s^{-1}$	BG-11 medium	shake-flasks	250 mL	
37	Oscillatoriaceae	*phormidium*	*Microcoleus vaginatus*	220.14	mg/g DW		25°C	cool-white fluorescent	40 μmol photons $m^{-2} s^{-1}$	BG-11 medium	shake-flasks	250 mL	
37	Oscillatoriaceae	*phormidium*	*Microcoleus vaginatus*	302.41	mg/g DW		25°C	cool-white fluorescent	80 μmol photons $m^{-2} s^{-1}$	BG-11 medium	shake-flasks	250 mL	
38	Scytonemataceae	*Scytonema*	*Scytonema tolypothrichoides*	23.82	mg/L		13°C	cool-white fluorescent	477 μmol photons $m^{-2} s^{-1}$	BG-11 medium			
38	Scytonemataceae	*Scytonema*	*Scytonema tolypothrichoides*	26.92	mg/L		13°C	cool-white fluorescent	238 μmol photons $m^{-2} s^{-1}$	BG-11 medium			
38	Scytonemataceae	*Scytonema*	*Scytonema tolypothrichoides*	26.75	mg/L		13°C	cool-white fluorescent	183 μmol photons $m^{-2} s^{-1}$	BG-11 medium			
38	Scytonemataceae	*Scytonema*	*Scytonema tolypothrichoides*	23.43	mg/L		13°C	cool-white fluorescent	91 μmol photons $m^{-2} s^{-1}$	BG-11 medium			
38	Scytonemataceae	*Scytonema*	*Scytonema tolypothrichoides*	51.29	mg/L		20°C	cool-white fluorescent	477 μmol photons $m^{-2} s^{-1}$	BG-11 medium			
38	Scytonemataceae	*Scytonema*	*Scytonema tolypothrichoides*	50	mg/L		20°C	cool-white fluorescent	238 μmol photons $m^{-2} s^{-1}$	BG-11 medium			
38	Scytonemataceae	*Scytonema*	*Scytonema tolypothrichoides*	30.57	mg/L		20°C	cool-white fluorescent	183 μmol photons $m^{-2} s^{-1}$	BG-11 medium			
38	Scytonemataceae	*Scytonema*	*Scytonema tolypothrichoides*	39.44	mg/L		20°C	cool-white fluorescent	91 μmol photons $m^{-2} s^{-1}$	BG-11 medium			
38	Scytonemataceae	*Scytonema*	*Scytonema tolypothrichoides*	90.21	mg/L		26°C	cool-white fluorescent	477 μmol photons $m^{-2} s^{-1}$	BG-11 medium			
38	Scytonemataceae	*Scytonema*	*Scytonema tolypothrichoides*	96.15	mg/L		26°C	cool-white fluorescent	238 μmol photons $m^{-2} s^{-1}$	BG-11 medium			
38	Scytonemataceae	*Scytonema*	*Scytonema tolypothrichoides*	107	mg/L		26°C	cool-white fluorescent	183 μmol photons $m^{-2} s^{-1}$	BG-11 medium			
38	Scytonemataceae	*Scytonema*	*Scytonema tolypothrichoides*	63.08	mg/L		26°C	cool-white fluorescent	91 μmol photons $m^{-2} s^{-1}$	BG-11 medium			
38	Scytonemataceae	*Scytonema*	*Scytonema tolypothrichoides*	114.3	mg/L		32°C	cool-white fluorescent	477 μmol photons $m^{-2} s^{-1}$	BG-11 medium			
38	Scytonemataceae	*Scytonema*	*Scytonema tolypothrichoides*	465.38	mg/L		32°C	cool-white fluorescent	238 μmol photons $m^{-2} s^{-1}$	BG-11 medium			
38	Scytonemataceae	*Scytonema*	*Scytonema tolypothrichoides*	256.05	mg/L		32°C	cool-white fluorescent	183 μmol photons $m^{-2} s^{-1}$	BG-11 medium			
38	Scytonemataceae	*Scytonema*	*Scytonema tolypothrichoides*	86.72	mg/L		32°C	cool-white fluorescent	91 μmol photons $m^{-2} s^{-1}$	BG-11 medium			
38	Scytonemataceae	*Scytonema*	*Scytonema tolypothrichoides*	57.02	mg/L		37°C	cool-white fluorescent	477 μmol photons $m^{-2} s^{-1}$	BG-11 medium			
38	Scytonemataceae	*Scytonema*	*Scytonema tolypothrichoides*	84.62	mg/L		37°C	cool-white fluorescent	238 μmol photons $m^{-2} s^{-1}$	BG-11 medium			
38	Scytonemataceae	*Scytonema*	*Scytonema tolypothrichoides*	38.22	mg/L		37°C	cool-white fluorescent	183 μmol photons $m^{-2} s^{-1}$	BG-11 medium			
38	Scytonemataceae	*Scytonema*	*Scytonema tolypothrichoides*	29.96	mg/L		37°C	cool-white fluorescent	91 μmol photons $m^{-2} s^{-1}$	BG-11 medium			
38	Scytonemataceae	*Tolypothrix*	*Tolypothrix bouteillei*	25.36	mg/L		13°C	cool-white fluorescent	477 μmol photons $m^{-2} s^{-1}$	BG-11 medium			
38	Scytonemataceae	*Tolypothrix*	*Tolypothrix bouteillei*	49.81	mg/L		13°C	cool-white fluorescent	238 μmol photons $m^{-2} s^{-1}$	BG-11 medium			
38	Scytonemataceae	*Tolypothrix*	*Tolypothrix bouteillei*	39.83	mg/L		13°C	cool-white fluorescent	183 μmol photons $m^{-2} s^{-1}$	BG-11 medium			
38	Scytonemataceae	*Tolypothrix*	*Tolypothrix bouteillei*	28.01	mg/L		13°C	cool-white fluorescent	91 μmol photons $m^{-2} s^{-1}$	BG-11 medium			
38	Scytonemataceae	*Tolypothrix*	*Tolypothrix bouteillei*	83.7	mg/L		20°C	cool-white fluorescent	477 μmol photons $m^{-2} s^{-1}$	BG-11 medium			
38	Scytonemataceae	*Tolypothrix*	*Tolypothrix bouteillei*	112.44	mg/L		20°C	cool-white fluorescent	238 μmol photons $m^{-2} s^{-1}$	BG-11 medium			
38	Scytonemataceae	*Tolypothrix*	*Tolypothrix bouteillei*	72.17	mg/L		20°C	cool-white fluorescent	183 μmol photons $m^{-2} s^{-1}$	BG-11 medium			
38	Scytonemataceae	*Tolypothrix*	*Tolypothrix bouteillei*	109.07	mg/L		20°C	cool-white fluorescent	91 μmol photons $m^{-2} s^{-1}$	BG-11 medium			
38	Scytonemataceae	*Tolypothrix*	*Tolypothrix bouteillei*	169.93	mg/L		26°C	cool-white fluorescent	477 μmol photons $m^{-2} s^{-1}$	BG-11 medium			
38	Scytonemataceae	*Tolypothrix*	*Tolypothrix bouteillei*	192.42	mg/L		26°C	cool-white fluorescent	238 μmol photons $m^{-2} s^{-1}$	BG-11 medium			
38	Scytonemataceae	*Tolypothrix*	*Tolypothrix bouteillei*	134.59	mg/L		26°C	cool-white fluorescent	183 μmol photons $m^{-2} s^{-1}$	BG-11 medium			
38	Scytonemataceae	*Tolypothrix*	*Tolypothrix bouteillei*	210.26	mg/L		26°C	cool-white fluorescent	91 μmol photons $m^{-2} s^{-1}$	BG-11 medium			
38	Scytonemataceae	*Tolypothrix*	*Tolypothrix bouteillei*	202.9	mg/L		32°C	cool-white fluorescent	477 μmol photons $m^{-2} s^{-1}$	BG-11 medium			
38	Scytonemataceae	*Tolypothrix*	*Tolypothrix bouteillei*	265.11	mg/L		32°C	cool-white fluorescent	238 μmol photons $m^{-2} s^{-1}$	BG-11 medium			
38	Scytonemataceae	*Tolypothrix*	*Tolypothrix bouteillei*	204.65	mg/L		32°C	cool-white fluorescent	183 μmol photons $m^{-2} s^{-1}$	BG-11 medium			
38	Scytonemataceae	*Tolypothrix*	*Tolypothrix bouteillei*	137.7	mg/L		32°C	cool-white fluorescent	91 μmol photons $m^{-2} s^{-1}$	BG-11 medium			
38	Scytonemataceae	*Tolypothrix*	*Tolypothrix bouteillei*	119.2	mg/L		37°C	cool-white fluorescent	477 μmol photons $m^{-2} s^{-1}$	BG-11 medium			
38	Scytonemataceae	*Tolypothrix*	*Tolypothrix bouteillei*	73.15	mg/L		37°C	cool-white fluorescent	238 μmol photons $m^{-2} s^{-1}$	BG-11 medium			
38	Scytonemataceae	*Tolypothrix*	*Tolypothrix bouteillei*	96.02	mg/L		37°C	cool-white fluorescent	183 μmol photons $m^{-2} s^{-1}$	BG-11 medium			
38	Scytonemataceae	*Tolypothrix*	*Tolypothrix bouteillei*	95.27	mg/L		37°C	cool-white fluorescent	91 μmol photons $m^{-2} s^{-1}$	BG-11 medium			
39	Cyanobacteriaceae	*Cyanobacterium*	*Cyanobacterium aponinum* PCC 10605	88.1	mg/g DW		29°C	cool-white fluorescent	15 μmol photons $m^{-2} s^{-1}$	BG-11 medium		250 mL	8
39	Cyanobacteriaceae	*Cyanobacterium*	*Cyanobacterium aponinum* PCC 10605	85.24	mg/g DW		29°C	cool-white fluorescent	40 μmol photons $m^{-2} s^{-1}$	BG-11 medium		250 mL	8
39	Cyanobacteriaceae	*Cyanobacterium*	*Cyanobacterium aponinum* PCC 10605	78.74	mg/g DW		29°C	cool-white fluorescent	70 μmol photons $m^{-2} s^{-1}$	BG-11 medium		250 mL	8
39	Cyanobacteriaceae	*Cyanobacterium*	*Cyanobacterium aponinum* PCC 10605	67.57	mg/g DW		29°C	cool-white fluorescent	100 μmol photons $m^{-2} s^{-1}$	BG-11 medium		250 mL	8
39	Cyanobacteriaceae	*Cyanobacterium*	*Cyanobacterium aponinum* PCC 10605	56.65	mg/g DW		29°C	cool-white fluorescent	150 μmol photons $m^{-2} s^{-1}$	BG-11 medium		250 mL	8
39	Cyanobacteriaceae	*Cyanobacterium*	*Cyanobacterium aponinum* PCC 10605	54.98	mg/g DW		29°C	cool-white fluorescent	300 μmol photons $m^{-2} s^{-1}$	BG-11 medium		250 mL	8
39	Cyanobacteriaceae	*Cyanobacterium*	*Cyanobacterium aponinum* PCC 10605	70.38	mg/g DW		29°C	cool-white fluorescent	500 μmol photons $m^{-2} s^{-1}$	BG-11 medium		250 mL	8
39	Cyanobacteriaceae	*Cyanobacterium*	*Cyanobacterium aponinum* PCC 10605	65.87	mg/g DW		29°C	cool-white fluorescent	650 μmol photons $m^{-2} s^{-1}$	BG-11 medium		250 mL	8
39	Cyanobacteriaceae	*Cyanobacterium*	*Cyanobacterium aponinum* PCC 10605	139.29	mg/g DW		28°C	cool-white fluorescent	35 μmol photons $m^{-2} s^{-1}$	BG-11 medium		250 mL	8
39	Cyanobacteriaceae	*Cyanobacterium*	*Cyanobacterium aponinum* PCC 10605	120.69	mg/g DW		35°C	cool-white fluorescent	35 μmol photons $m^{-2} s^{-1}$	BG-11 medium		250 mL	8
39	Cyanobacteriaceae	*Cyanobacterium*	*Cyanobacterium aponinum* PCC 10605	86.03	mg/g DW		40°C	cool-white fluorescent	35 μmol photons $m^{-2} s^{-1}$	BG-11 medium		250 mL	8
40	Chrococcaceae	*Synechocystis*	*Microcystis aeruginosa* FACHB 927	100	%	0.26 mg/L $NaNO_3$	25°C	cool-white fluorescent	35 μmol photons $m^{-2} s^{-1}$	BG-11 medium		1000 mL	
40	Chrococcaceae	*Synechocystis*	*Microcystis aeruginosa* FACHB 927	29	%	0.26 mg/L $NaNO_3$	25°C	cool-white fluorescent	80 μmol photons $m^{-2} s^{-1}$	BG-11 medium		1000 mL	
40	Chrococcaceae	*Synechocystis*	*Microcystis aeruginosa* FACHB 927	51.47	%	2.55 mg/L $NaNO_3$	25°C	cool-white fluorescent	35 μmol photons $m^{-2} s^{-1}$	BG-11 medium		1000 mL	
40	Chrococcaceae	*Synechocystis*	*Microcystis aeruginosa* FACHB 927	73.22	%	2.55 mg/L $NaNO_3$	25°C	cool-white fluorescent	80 μmol photons $m^{-2} s^{-1}$	BG-11 medium		1000 mL	
40	Chrococcaceae	*Synechocystis*	*Microcystis aeruginosa* FACHB 927	56.02	%	0.26 mg/L $NaNO_3$	30°C	cool-white fluorescent	35 μmol photons $m^{-2} s^{-1}$	BG-11 medium		1000 mL	
40	Chrococcaceae	*Synechocystis*	*Microcystis aeruginosa* FACHB 927	24.2	%	0.26 mg/L $NaNO_3$	30°C	cool-white fluorescent	80 μmol photons $m^{-2} s^{-1}$	BG-11 medium		1000 mL	
40	Chrococcaceae	*Synechocystis*	*Microcystis aeruginosa* FACHB 927	51.3	%	2.55 mg/L $NaNO_3$	30°C	cool-white fluorescent	35 μmol photons $m^{-2} s^{-1}$	BG-11 medium		1000 mL	
40	Chrococcaceae	*Synechocystis*	*Microcystis aeruginosa* FACHB 927	43.6	%	2.55 mg/L $NaNO_3$	30°C	cool-white fluorescent	80 μmol photons $m^{-2} s^{-1}$	BG-11 medium		1000 mL	
41	Chrococcaceae	*Synechocystis*	*Microcystis aeruginosa Kützing* PCC 7806	1.56	pg/cell		25°C	cool-white fluorescent	5 μmol photons $m^{-2} s^{-1}$	BG-11 medium			
41	Chrococcaceae	*Synechocystis*	*Microcystis aeruginosa Kützing* PCC 7806	1.51	pg/cell		25°C	cool-white fluorescent	40 μmol photons $m^{-2} s^{-1}$	BG-11 medium			
41	Chrococcaceae	*Synechocystis*	*Microcystis aeruginosa Kützing* PCC 7806	2.43	pg/cell		25°C	cool-white fluorescent	100 μmol photons $m^{-2} s^{-1}$	BG-11 medium			
41	Chrococcaceae	*Synechocystis*	*Microcystis aeruginosa Kützing* PCC 7806	1.59	pg/cell		15°C	cool-white fluorescent	40 μmol photons $m^{-2} s^{-1}$	BG-11 medium			
41	Chrococcaceae	*Synechocystis*	*Microcystis aeruginosa Kützing* PCC 7806	1.63	pg/cell		25°C	cool-white fluorescent	40 μmol photons $m^{-2} s^{-1}$	BG-11 medium			
41	Chrococcaceae	*Synechocystis*	*Microcystis aeruginosa Kützing* PCC 7806	1.66	pg/cell		30°C	cool-white fluorescent	40 μmol photons $m^{-2} s^{-1}$	BG-11 medium			
41	Chrococcaceae	*Synechocystis*	*Microcystis aeruginosa Kützing* PCC 7806	1.76	pg/cell	ck P	25°C	cool-white fluorescent	40 μmol photons $m^{-2} s^{-1}$	BG-11 medium			
41	Chrococcaceae	*Synechocystis*	*Microcystis aeruginosa Kützing* PCC 7806	1.75	pg/cell	no P	25°C	cool-white fluorescent	40 μmol photons $m^{-2} s^{-1}$	BG-11 medium			
41	Chrococcaceae	*Synechocystis*	*Microcystis aeruginosa Kützing* PCC 7806	2.03	pg/cell	no N	25°C	cool-white fluorescent	40 μmol photons $m^{-2} s^{-1}$	BG-11 medium			
42	Chrococcaceae	*Synechocystis*	*Microcystis aeruginosa* FACHB 912	0.35	pg/cell		25°C		100 μmol photons $m^{-2} s^{-1}$	BG11 medium			
42	Chrococcaceae	*Synechocystis*	*Microcystis aeruginosa* FACHB 912	0.54	pg/cell		25°C	UVB radiation	100 μmol photons $m^{-2} s^{-1}$	BG11 medium			
42	Chrococcaceae	*Synechocystis*	*Microcystis aeruginosa* FACHB 912	0.73	pg/cell		25°C		200 μmol photons $m^{-2} s^{-1}$	BG11 medium			
42	Chrococcaceae	*Synechocystis*	*Microcystis aeruginosa* FACHB 912	0.4	pg/cell		25°C	UVB radiation	200 μmol photons $m^{-2} s^{-1}$	BG11 medium			
42	Chrococcaceae	*Synechocystis*	*Microcystis aeruginosa* FACHB 912	0.69	pg/cell		25°C		300 μmol photons $m^{-2} s^{-1}$	BG11 medium			
42	Chrococcaceae	*Synechocystis*	*Microcystis aeruginosa* FACHB 912	1.4	pg/cell		25°C	UVB radiation	300 μmol photons $m^{-2} s^{-1}$	BG11 medium			
42	Chrococcaceae	*Synechocystis*	*Microcystis aeruginosa* FACHB 912	1.4	pg/cell		25°C		400 μmol photons $m^{-2} s^{-1}$	BG11 medium			
42	Chrococcaceae	*Synechocystis*	*Microcystis aeruginosa* FACHB 912	1.01	pg/cell		25°C	UVB radiation	400 μmol photons $m^{-2} s^{-1}$	BG11 medium			
42	Chrococcaceae	*Synechocystis*	*Microcystis aeruginosa* FACHB469	1.22	pg/cell		25°C		100 μmol photons $m^{-2} s^{-1}$	BG11 medium			
42	Chrococcaceae	*Synechocystis*	*Microcystis aeruginosa* FACHB469	0.28	pg/cell		25°C	UVB radiation	100 μmol photons $m^{-2} s^{-1}$	BG11 medium			
42	Chrococcaceae	*Synechocystis*	*Microcystis aeruginosa* FACHB469	0.39	pg/cell		25°C		200 μmol photons $m^{-2} s^{-1}$	BG11 medium			
42	Chrococcaceae	*Synechocystis*	*Microcystis aeruginosa* FACHB469	0.32	pg/cell		25°C	UVB radiation	200 μmol photons $m^{-2} s^{-1}$	BG11 medium			
42	Chrococcaceae	*Synechocystis*	*Microcystis aeruginosa* FACHB469	0.42	pg/cell		25°C		300 μmol photons $m^{-2} s^{-1}$	BG11 medium			
42	Chrococcaceae	*Synechocystis*	*Microcystis aeruginosa* FACHB469	0.6	pg/cell		25°C	UVB radiation	300 μmol photons $m^{-2} s^{-1}$	BG11 medium			
42	Chrococcaceae	*Synechocystis*	*Microcystis aeruginosa* FACHB469	0.89	pg/cell		25°C		400 μmol photons $m^{-2} s^{-1}$	BG11 medium			
42	Chrococcaceae	*Synechocystis*	*Microcystis aeruginosa* FACHB469	1.1	pg/cell		25°C	UVB radiation	400 μmol photons $m^{-2} s^{-1}$	BG11 medium			
45	Nostoc	*Nostoc*	*Nostoc punctiforme* ATCC 29133	0.18	mg/mg Chl a				40 μmol photons $m^{-2} s^{-1}$				
45	Nostoc	*Nostoc*	*Nostoc punctiforme* ATCC 29133	0.21	mg/mg Chl a			UVA	40 μmol photons $m^{-2} s^{-1}$				
45	Scytonemataceae	*Scytonema*	*scytonemin-deficient* mutant, SCY59	0.23	mg/mg Chl a				40 μmol photons $m^{-2} s^{-1}$				
45	Scytonemataceae	*Scytonema*	*scytonemin-deficient* mutant, SCY59	0.54	mg/mg Chl a			UVA	40 μmol photons $m^{-2} s^{-1}$				
46	Nostoc	*Nostoc*	*Nostoc flagelliforme*	224.92	mg/g DW		25°C	cool-white fluorescent	60 μmol photons $m^{-2} s^{-1}$	BG-11 medium	shake-flasks	500 mL	9
46	Nostoc	*Nostoc*	*Nostoc flagelliforme*	341.19	mg/g DW		25°C	red	60 μmol photons $m^{-2} s^{-1}$	BG-11 medium	shake-flasks	500 mL	9
46	Nostoc	*Nostoc*	*Nostoc flagelliforme*	281.73	mg/g DW		25°C	blue	60 μmol photons $m^{-2} s^{-1}$	BG-11 medium	shake-flasks	500 mL	9
46	Nostoc	*Nostoc*	*Nostoc flagelliforme*	287.7	mg/g DW		25°C	red and blue	60 μmol photons $m^{-2} s^{-1}$	BG-11 medium	shake-flasks	500 mL	9
47	Nostoc	*Nostoc*	*Nostoc flagelliforme*	44.44	mg/g DW			red	60 μmol photons $m^{-2} s^{-1}$			500 mL	7
47	Nostoc	*Nostoc*	*Nostoc flagelliforme*	47.39	mg/g DW			blue	60 μmol photons $m^{-2} s^{-1}$			500 mL	7
47	Nostoc	*Nostoc*	*Nostoc flagelliforme*	31.43	mg/g DW			white	60 μmol photons $m^{-2} s^{-1}$			500 mL	7
47	Nostoc	*Nostoc*	*Nostoc flagelliforme*	34.24	mg/g DW			green	60 μmol photons $m^{-2} s^{-1}$			500 mL	7
47	Nostoc	*Nostoc*	*Nostoc flagelliforme*	29.72	mg/g DW			yellow	60 μmol photons $m^{-2} s^{-1}$			500 mL	7
47	Nostoc	*Nostoc*	*Nostoc flagelliforme*	42.42	mg/g DW			purple	60 μmol photons $m^{-2} s^{-1}$			500 mL	7
48	Oscillatoriaceae	*Lyngbya*	*Lyngbya stagnina*	20	mg/L		21°C	cool-white fluorescent	45 μmol photons $m^{-2} s^{-1}$	Chu-10 medium			
48	Oscillatoriaceae	*Lyngbya*	*Lyngbya stagnina*	42	mg/L		28°C	cool-white fluorescent	45 μmol photons $m^{-2} s^{-1}$	Chu-10 medium	shake-flasks		
48	Oscillatoriaceae	*Lyngbya*	*Lyngbya stagnina*	49.6	mg/L		35°C	cool-white fluorescent	45 μmol photons $m^{-2} s^{-1}$	Chu-10 medium	shake-flasks		
48	Oscillatoriaceae	*Lyngbya*	*Lyngbya stagnina*	29.2	mg/L		28°C	cool-white fluorescent	45 μmol photons $m^{-2} s^{-1}$	Chu-10 medium	shake-flasks		
48	Oscillatoriaceae	*Lyngbya*	*Lyngbya stagnina*	54	mg/L	1.12 mM $CaCl_2$	28°C	cool-white fluorescent	45 μmol photons $m^{-2} s^{-1}$	Chu-10 medium	shake-flasks		
48	Oscillatoriaceae	*Lyngbya*	*Lyngbya stagnina*	33.6	mg/L	3 mM $CaCl_2$	28°C	cool-white fluorescent	45 μmol photons $m^{-2} s^{-1}$	Chu-10 medium	shake-flasks		
48	Oscillatoriaceae	*Lyngbya*	*Lyngbya stagnina*	25.6	mg/L	3.75 mM $CaCl_2$	28°C	cool-white fluorescent	45 μmol photons $m^{-2} s^{-1}$	Chu-10 medium	shake-flasks		
48	Oscillatoriaceae	*Lyngbya*	*Lyngbya stagnina*	28.8	mg/L	8.5 mM NaCl	28°C	cool-white fluorescent	45 μmol photons $m^{-2} s^{-1}$	Chu-10 medium	shake-flasks		
48	Oscillatoriaceae	*Lyngbya*	*Lyngbya stagnina*	48.8	mg/L	17 mM NaCl	28°C	cool-white fluorescent	45 μmol photons $m^{-2} s^{-1}$	Chu-10 medium	shake-flasks		
48	Oscillatoriaceae	*Lyngbya*	*Lyngbya stagnina*	88	mg/L	85 mM NaCl	28°C	cool-white fluorescent	45 μmol photons $m^{-2} s^{-1}$	Chu-10 medium	shake-flasks		
48	Oscillatoriaceae	*Lyngbya*	*Lyngbya stagnina*	84	mg/L	170 mM NaCl	28°C	cool-white fluorescent	45 μmol photons $m^{-2} s^{-1}$	Chu-10 medium	shake-flasks		
48	Oscillatoriaceae	*Lyngbya*	*Lyngbya stagnina*	55.6	mg/L	340 mM NaCl	28°C	cool-white fluorescent	45 μmol photons $m^{-2} s^{-1}$	Chu-10 medium	shake-flasks		
48	Oscillatoriaceae	*Lyngbya*	*Lyngbya stagnina*	35.6	mg/L	5 mM NO_3^-	28°C	cool-white fluorescent	45 μmol photons $m^{-2} s^{-1}$	Chu-10 medium	shake-flasks		
48	Oscillatoriaceae	*Lyngbya*	*Lyngbya stagnina*	50.8	mg/L	2 mM NO_3^-	28°C	cool-white fluorescent	45 μmol photons $m^{-2} s^{-1}$	Chu-10 medium	shake-flasks		
48	Oscillatoriaceae	*Lyngbya*	*Lyngbya stagnina*	78	mg/L	0.5 mM NO_3^-	28°C	cool-white fluorescent	45 μmol photons $m^{-2} s^{-1}$	Chu-10 medium	shake-flasks		
48	Oscillatoriaceae	*Lyngbya*	*Lyngbya stagnina*	35.6	mg/L	0.1 mM NO_3^-	28°C	cool-white fluorescent	45 μmol photons $m^{-2} s^{-1}$	Chu-10 medium	shake-flasks		
49	Nostoc	*Nostoc*	*Nostoc* PCC 7936	310.23	mg/L	CO_2	30°C		70 μmol photons $m^{-2} s^{-1}$	BG11o medium (BG11 without $NaNO_3$)			8.1
49	Nostoc	*Nostoc*	*Nostoc* PCC 7936	248.14	mg/L		30°C		70 μmol photons $m^{-2} s^{-1}$	BG11o medium (BG11 without $NaNO_3$)			8.1
49	Nostoc	*Nostoc*	*Nostoc* PCC 7936	377.92	mg/L	CO_2	30°C		70 μmol photons $m^{-2} s^{-1}$	BG11o medium (BG11 without $NaNO_3$)			7.5
49	Nostoc	*Nostoc*	*Nostoc* PCC 7936	184.2	mg/L		30°C		70 μmol photons $m^{-2} s^{-1}$	BG11o medium (BG11 without $NaNO_3$)			7.5
49	Nostoc	*Nostoc*	*Nostoc* PCC 7936	555.37	mg/L	$NaNO_3$+CO_2	30°C		70 μmol photons $m^{-2} s^{-1}$	BG11 medium			8.1
49	Nostoc	*Nostoc*	*Nostoc* PCC 7936	387.34	mg/L	$NaNO_3$	30°C		70 μmol photons $m^{-2} s^{-1}$	BG11 medium			8.1
49	Nostoc	*Nostoc*	*Nostoc* PCC 7936	282.92	mg/L	CO_2	35°C		70 μmol photons $m^{-2} s^{-1}$	BG11 medium			8.1
49	Nostoc	*Nostoc*	*Nostoc* PCC 7936	188.56	mg/L		35°C		70 μmol photons $m^{-2} s^{-1}$	BG11 medium			8.1
49	Nostoc	*Nostoc*	*Nostoc* PCC 7936	388.56	mg/L	CO_2	35°C		70 μmol photons $m^{-2} s^{-1}$	BG11o medium (BG11 without $NaNO_3$)			7.5
49	Nostoc	*Nostoc*	*Nostoc* PCC 7936	218.75	mg/L		35°C		70 μmol photons $m^{-2} s^{-1}$	BG11o medium (BG11 without $NaNO_3$)			7.5
49	Nostoc	*Nostoc*	*Nostoc* PCC 7936	254.58	mg/L	$NaNO_3$	35°C		70 μmol photons $m^{-2} s^{-1}$	BG11 medium			8.1
49	Nostoc	*Nostoc*	*Nostoc* PCC 7936	254.58	mg/L		35°C		70 μmol photons $m^{-2} s^{-1}$	BG11 medium			8.1
49	Nostoc	*Nostoc*	*Nostoc* PCC 7936	441.53	mg/L	$NaNO_3$+CO_2	35°C		70 μmol photons $m^{-2} s^{-1}$	BG11 medium			7.5
49	Nostoc	*Nostoc*	*Nostoc* PCC 7936	208.41	mg/L	$NaNO_3$	35°C		70 μmol photons $m^{-2} s^{-1}$	BG11 medium			7.5
50	Nostoc	*Nostoc*	*Nostoc* sp. BTA97	0.5	mg/mL	1.5 g/L $NaNO_3$	28°C		60 μmol photons $m^{-2} s^{-1}$				
50	Nostoc	*Nostoc*	*Nostoc* sp. BTA97	0.65	mg/mL	0.75 g/L $NaNO_3$	28°C		60 μmol photons $m^{-2} s^{-1}$				
50	Nostoc	*Nostoc*	*Nostoc* sp. BTA97	1.62	mg/mL	0 g/L $NaNO_3$	28°C		60 μmol photons $m^{-2} s^{-1}$				
50	Nostoc	*Anabaena*	*Anabaena* sp. BTA990	0.5	mg/mL	1.5 g/L $NaNO_3$	28°C		60 μmol photons $m^{-2} s^{-1}$				
50	Nostoc	*Anabaena*	*Anabaena* sp. BTA990	0.93	mg/mL	0.75 g/L $NaNO_3$	28°C		60 μmol photons $m^{-2} s^{-1}$				
50	Nostoc	*Anabaena*	*Anabaena* sp. BTA990	1.23	mg/mL	0 g/L $NaNO_3$	28°C		60 μmol photons $m^{-2} s^{-1}$				
50	Nostoc	*Nostoc*	*Nostoc* sp. BTA97	0.92	mg/mL		28°C		60 μmol photons $m^{-2} s^{-1}$				6.6
50	Nostoc	*Nostoc*	*Nostoc* sp. BTA97	1.08	mg/mL		28°C		60 μmol photons $m^{-2} s^{-1}$				7
50	Nostoc	*Nostoc*	*Nostoc* sp. BTA97	1.14	mg/mL		28°C		60 μmol photons $m^{-2} s^{-1}$				7.5
50	Nostoc	*Nostoc*	*Nostoc* sp. BTA97	1.83	mg/mL		28°C		60 μmol photons $m^{-2} s^{-1}$				8
50	Nostoc	*Anabaena*	*Anabaena* sp. BTA990	0.76	mg/mL		28°C		60 μmol photons $m^{-2} s^{-1}$				6.6
50	Nostoc	*Anabaena*	*Anabaena* sp. BTA990	0.89	mg/mL		28°C		60 μmol photons $m^{-2} s^{-1}$				7
50	Nostoc	*Anabaena*	*Anabaena* sp. BTA990	1.01	mg/mL		28°C		60 μmol photons $m^{-2} s^{-1}$				7.5
50	Nostoc	*Anabaena*	*Anabaena* sp. BTA990	1.68	mg/mL		28°C		60 μmol photons $m^{-2} s^{-1}$				8
51	Nostoc	*Nostoc*	*Nostoc flagelliforme*	0.46	ug/ug DW/day		25°C		60 μmol photons $m^{-2} s^{-1}$	BG-11 medium			7.5
51	Nostoc	*Nostoc*	*Nostoc flagelliforme*	1.32	ug/ug DW/day		25°C		60 μmol photons $m^{-2} s^{-1}$	BG-11 medium			9
52	Oscillatoriaceae	*Spirulina*	*Spirulina platensis*	1.5	g/L		30°C						5
52	Oscillatoriaceae	*Spirulina*	*Spirulina platensis*	2.2	g/L		30°C						5.5
52	Oscillatoriaceae	*Spirulina*	*Spirulina platensis*	3.45	g/L		30°C						6
52	Oscillatoriaceae	*Spirulina*	*Spirulina platensis*	4.24	g/L		30°C						6.5
52	Oscillatoriaceae	*Spirulina*	*Spirulina platensis*	3.83	g/L		30°C						7
52	Oscillatoriaceae	*Spirulina*	*Spirulina platensis*	3.41	g/L		30°C						7.5
52	Oscillatoriaceae	*Spirulina*	*Spirulina platensis*	2.62	g/L		30°C						8
54	Trichodesmium	*Trichodesmium*	*Trichodesmium erythraeum* IMS101	51.71	μg/L	pH=8.0, CO_2=400 ppm	26°C		80 μmol photons $m^{-2} s^{-1}$	YBCII medium			
54	Trichodesmium	*Trichodesmium*	*Trichodesmium erythraeum* IMS101	176.22	μg/L	pH=7.8, CO_2=1000 ppm	26°C		80 μmol photons $m^{-2} s^{-1}$	YBCII medium			
55	Trichodesmium	*Trichodesmium*	*Trichodesmium erythraeum* IMS101	180.00	μg/L	pH=8.0, CO_2=1000 ppm	26°C		80 μmol photons $m^{-2} s^{-1}$	YBCII medium			
55	Trichodesmium	*Trichodesmium*	*Trichodesmium erythraeum* IMS101	52.67	μg/L	pH=8.0, CO_2=400 ppm	26°C		80 μmol photons $m^{-2} s^{-1}$	YBCII medium			
55	Trichodesmium	*Trichodesmium*	*Trichodesmium erythraeum* IMS101	133	μg/L	pH=7.8, CO_2=1000 ppm	26°C		80 μmol photons $m^{-2} s^{-1}$	YBCII medium			
55	Trichodesmium	*Trichodesmium*	*Trichodesmium erythraeum* IMS101	43.33	μg/L	pH=7.8, CO_2=400 ppm	26°C		80 μmol photons $m^{-2} s^{-1}$	YBCII medium			
56	Cyanobiaceae	*Cyanothece*	*Cyanothece* ATCC 51142	0.38	g% w/v		27°C	cool-white fluorescent	55 μmol photons $m^{-2} s^{-1}$	ASN III medium			5
56	Cyanobiaceae	*Cyanothece*	*Cyanothece* ATCC 51142	0.54	g% w/v		27°C	cool-white fluorescent	55 μmol photons $m^{-2} s^{-1}$	ASN III medium			7
56	Cyanobiaceae	*Cyanothece*	*Cyanothece* ATCC 51142	0.81	g% w/v		27°C	cool-white fluorescent	55 μmol photons $m^{-2} s^{-1}$	ASN III medium			9
56	Cyanobiaceae	*Cyanothece*	*Cyanothece* ATCC 51142	0.22	g% w/v		27°C	cool-white fluorescent	55 μmol photons $m^{-2} s^{-1}$	ASN III medium			11
56	Cyanobiaceae	*Cyanothece*	*Cyanothece* ATCC 51142	0.37	g% w/v	0 % NaCl	27°C	cool-white fluorescent	55 μmol photons $m^{-2} s^{-1}$	ASN III medium			7
56	Cyanobiaceae	*Cyanothece*	*Cyanothece* ATCC 51142	1.79	g% w/v	1.5 % NaCl	27°C	cool-white fluorescent	55 μmol photons $m^{-2} s^{-1}$	ASN III medium			7
56	Cyanobiaceae	*Cyanothece*	*Cyanothece* ATCC 51142	2.11	g% w/v	2.5 % NaCl	27°C	cool-white fluorescent	55 μmol photons $m^{-2} s^{-1}$	ASN III medium			7
56	Cyanobiaceae	*Cyanothece*	*Cyanothece* ATCC 51142	3.14	g% w/v	3.5 % NaCl	27°C	cool-white fluorescent	55 μmol photons $m^{-2} s^{-1}$	ASN III medium			7
56	Cyanobiaceae	*Cyanothece*	*Cyanothece* ATCC 51142	4.09	g% w/v	4.5% NaCl	27°C	cool-white fluorescent	55 μmol photons $m^{-2} s^{-1}$	ASN III medium			7
56	Cyanobiaceae	*Cyanothece*	*Cyanothece* ATCC 51142	1.58	g% w/v	$NaNO_3$	27°C	cool-white fluorescent	55 μmol photons $m^{-2} s^{-1}$	ASN III medium			7
56	Cyanobiaceae	*Cyanothece*	*Cyanothece* ATCC 51142	1.08	g% w/v	KNO_3	27°C	cool-white fluorescent	55 μmol photons $m^{-2} s^{-1}$	ASN III medium			7
56	Cyanobiaceae	*Cyanothece*	*Cyanothece* ATCC 51142	0.69	g% w/v	N_2 (air)	27°C	cool-white fluorescent	55 μmol photons $m^{-2} s^{-1}$	ASN III medium			7
59	Oscillatoriaceae	*Spirulina*	*Spirulina* sp.	9	mg/L		25°C	cool-white fluorescent	27 μmol photons $m^{-2} s^{-1}$	Alkaline	shake-flasks	1500 mL	10
59	Oscillatoriaceae	*Spirulina*	*Spirulina* sp.	40	mg/L	1.25 g/L $NaNO_3$	25°C	cool-white fluorescent	27 μmol photons $m^{-2} s^{-1}$	Alkaline	shake-flasks	1500 mL	10
59	Oscillatoriaceae	*Spirulina*	*Spirulina* sp.	7	mg/L	4.0 g/L $NaNO_3$	25°C	cool-white fluorescent	27 μmol photons $m^{-2} s^{-1}$	Alkaline	shake-flasks	1500 mL	10
59	Oscillatoriaceae	*Spirulina*	*Spirulina* sp.	11.7	mg/L	1.0 g/L K_2HPO_4	25°C	cool-white fluorescent	27 μmol photons $m^{-2} s^{-1}$	Alkaline	shake-flasks	1500 mL	10
59	Oscillatoriaceae	*Spirulina*	*Spirulina* sp.	30	mg/L	no K_2HPO_4	25°C	cool-white fluorescent	27 μmol photons $m^{-2} s^{-1}$	Alkaline	shake-flasks	1500 mL	10
59	Oscillatoriaceae	*phormidium*	*Phormidium* sp.	30	mg/L		25°C	cool-white fluorescent	27 μmol photons $m^{-2} s^{-1}$	BG-11 medium	shake-flasks	1500 mL	8.1
59	Oscillatoriaceae	*phormidium*	*Phormidium* sp.	9.7	mg/L	3 g/L $NaNO_3$	25°C	cool-white fluorescent	27 μmol photons $m^{-2} s^{-1}$	BG-11 medium	shake-flasks	1500 mL	8.1
59	Oscillatoriaceae	*phormidium*	*Phormidium* sp.	11	mg/L	0.12 g/L K_2HPO_4	25°C	cool-white fluorescent	27 μmol photons $m^{-2} s^{-1}$	BG-11 medium	shake-flasks	1500 mL	8.1
59	Oscillatoriaceae	*phormidium*	*Phormidium* sp.	2.9	mg/L	no K_2HPO_4	25°C	cool-white fluorescent	27 μmol photons $m^{-2} s^{-1}$	BG-11 medium	shake-flasks	1500 mL	8.1
59	Nostoc	*Anabaena*	*Anabaena torulosa*	22	mg/L		25°C	cool-white fluorescent	27 μmol photons $m^{-2} s^{-1}$	BG-11 medium	shake-flasks	1500 mL	8.1
59	Nostoc	*Anabaena*	*Anabaena torulosa*	10.2	mg/L	3 g/L $NaNO_3$	25°C	cool-white fluorescent	27 μmol photons $m^{-2} s^{-1}$	BG-11 medium	shake-flasks	1500 mL	8.1
59	Nostoc	*Anabaena*	*Anabaena torulosa*	24	mg/L	0.12 g/L K_2HPO_4	25°C	cool-white fluorescent	27 μmol photons $m^{-2} s^{-1}$	BG-11 medium	shake-flasks	1500 mL	8.1
59	Nostoc	*Anabaena*	*Anabaena torulosa*	13.6	mg/L	no K_2HPO_4	25°C	cool-white fluorescent	27 μmol photons $m^{-2} s^{-1}$	BG-11 medium	shake-flasks	1500 mL	8.1
59	Nostoc	*Anabaena*	*Anabaena* WSAF	56	mg/L		25°C	cool-white fluorescent	27 μmol photons $m^{-2} s^{-1}$	BG-11 medium	shake-flasks	1500 mL	8.1
59	Nostoc	*Anabaena*	*Anabaena* WSAF	22	mg/L	3 g/L $NaNO_3$	25°C	cool-white fluorescent	27 μmol photons $m^{-2} s^{-1}$	BG-11 medium	shake-flasks	1500 mL	8.1
59	Nostoc	*Anabaena*	*Anabaena* WSAF	46	mg/L	0.12 g/L K_2HPO_4	26°C	cool-white fluorescent	27 μmol photons $m^{-2} s^{-1}$	BG-11 medium	shake-flasks	1500 mL	8.1
59	Nostoc	*Anabaena*	*Anabaena* WSAF	4	mg/L	no K_2HPO_4	26°C	cool-white fluorescent	27 μmol photons $m^{-2} s^{-1}$	BG-11 medium	shake-flasks	1500 mL	8.1
60	Nostoc	*Nostoc*	*Nostoc flagelliforme*	44	mg/L	1 mg/L $NaNO_3$	25°C		60 μmol photons $m^{-2} s^{-1}$	BG11 medium		200 mL	
60	Nostoc	*Nostoc*	*Nostoc flagelliforme*	73	mg/L	1.5 mg/L $NaNO_3$	25°C		60 μmol photons $m^{-2} s^{-1}$	BG11 medium		200 mL	
60	Nostoc	*Nostoc*	*Nostoc flagelliforme*	100	mg/L	2.5 mg/L $NaNO_3$	25°C		60 μmol photons $m^{-2} s^{-1}$	BG11 medium		200 mL	
60	Nostoc	*Nostoc*	*Nostoc flagelliforme*	62	mg/L	3.5 mg/L $NaNO_3$	25°C		60 μmol photons $m^{-2} s^{-1}$	BG11 medium		200 mL	
60	Nostoc	*Nostoc*	*Nostoc flagelliforme*	71	mg/L	0.024mg/L K_2HPO_4	25°C		60 μmol photons $m^{-2} s^{-1}$	BG11 medium		200 mL	
60	Nostoc	*Nostoc*	*Nostoc flagelliforme*	76	mg/L	0.238mg/L K_2HPO_4	25°C		60 μmol photons $m^{-2} s^{-1}$	BG11 medium		200 mL	
60	Nostoc	*Nostoc*	*Nostoc flagelliforme*	97	mg/L	0.956mg/L K_2HPO_4	25°C		60 μmol photons $m^{-2} s^{-1}$	BG11 medium		200 mL	
60	Nostoc	*Nostoc*	*Nostoc flagelliforme*	93	mg/L	1.912mg/L K_2HPO_4	25°C		60 μmol photons $m^{-2} s^{-1}$	BG11 medium		200 mL	
61	Nostoc	*Nostoc*	*Nostoc muscorum*	45.82	mg/L	0 g/LK_2HPO_4	25°C		50 μmol photons $m^{-2} s^{-1}$	BG11o medium (BG11 without $NaNO_3$)			
61	Nostoc	*Nostoc*	*Nostoc muscorum*	57.94	mg/L	0.01 g/LK_2HPO_4	25°C		50 μmol photons $m^{-2} s^{-1}$	BG11o medium (BG11 without $NaNO_3$)			
61	Nostoc	*Nostoc*	*Nostoc muscorum*	72.27	mg/L	0.05 g/LK_2HPO_4	25°C		50 μmol photons $m^{-2} s^{-1}$	BG11o medium (BG11 without $NaNO_3$)			
61	Nostoc	*Nostoc*	*Nostoc muscorum*	73.64	mg/L	0.09 g/LK_2HPO_4	25°C		50 μmol photons $m^{-2} s^{-1}$	BG11o medium (BG11 without $NaNO_3$)			
61	Nostoc	*Nostoc*	*Nostoc muscorum*	50.68	mg/L	0.12 g/LK_2HPO_4	25°C		50 μmol photons $m^{-2} s^{-1}$	BG11o medium (BG11 without $NaNO_3$)			
61	Nostoc	*Nostoc*	*Nostoc muscorum*	72.27	mg/L	0 g/L $MgSO_4$	25°C		50 μmol photons $m^{-2} s^{-1}$	BG11o medium (BG11 without $NaNO_3$)			
61	Nostoc	*Nostoc*	*Nostoc muscorum*	73.64	mg/L	0.01 g/L $MgSO_4$	25°C		50 μmol photons $m^{-2} s^{-1}$	BG11o medium (BG11 without $NaNO_3$)			
61	Nostoc	*Nostoc*	*Nostoc muscorum*	116.32	mg/L	0.08 g/L $MgSO_4$	25°C		50 μmol photons $m^{-2} s^{-1}$	BG11o medium (BG11 without $NaNO_3$)			
61	Nostoc	*Nostoc*	*Nostoc muscorum*	61.47	mg/L	0.15 g/L $MgSO_4$	25°C		50 μmol photons $m^{-2} s^{-1}$	BG11o medium (BG11 without $NaNO_3$)			
61	Nostoc	*Nostoc*	*Nostoc muscorum*	43.38	mg/L	0.197 g/L $MgSO_4$	25°C		50 μmol photons $m^{-2} s^{-1}$	BG11o medium (BG11 without $NaNO_3$)			
61	Nostoc	*Nostoc*	*Nostoc muscorum*	28.87	mg/L	0 g/L NaCl	25°C		50 μmol photons $m^{-2} s^{-1}$	BG11o medium (BG11 without $NaNO_3$)			
61	Nostoc	*Nostoc*	*Nostoc muscorum*	57.94	mg/L	0.003 g/L NaCl	25°C		50 μmol photons $m^{-2} s^{-1}$	BG11o medium (BG11 without $NaNO_3$)			
61	Nostoc	*Nostoc*	*Nostoc muscorum*	115.8	mg/L	0.0515 g/L NaCl	25°C		50 μmol photons $m^{-2} s^{-1}$	BG11o medium (BG11 without $NaNO_3$)			
61	Nostoc	*Nostoc*	*Nostoc muscorum*	73.64	mg/L	0.1 g/L NaCl	25°C		50 μmol photons $m^{-2} s^{-1}$	BG11o medium (BG11 without $NaNO_3$)			
61	Nostoc	*Nostoc*	*Nostoc muscorum*	67.07	mg/L	0.133 g/L NaCl	25°C		50 μmol photons $m^{-2} s^{-1}$	BG11o medium (BG11 without $NaNO_3$)			
62	Chrococcaceae	*Synechocystis*	*Synechocystis* sp. BASO444	500.58	mg/L	0 mol/L NaCl	25°C		220 μmol photons $m^{-2} s^{-1}$	BG-11 medium			
62	Chrococcaceae	*Synechocystis*	*Synechocystis* sp. BASO444	547.9	mg/L	0.2 mol/L NaCl	25°C		220 μmol photons $m^{-2} s^{-1}$	BG-11 medium			
62	Chrococcaceae	*Synechocystis*	*Synechocystis* sp. BASO444	639.01	mg/L	0.4 mol/L NaCl	25°C		220 μmol photons $m^{-2} s^{-1}$	BG-11 medium			
62	Chrococcaceae	*Synechocystis*	*Synechocystis* sp. BASO511	471.39	mg/L	0 mol/L NaCl	25°C		220 μmol photons $m^{-2} s^{-1}$	BG-11 medium			
62	Chrococcaceae	*Synechocystis*	*Synechocystis* sp. BASO511	528.11	mg/L	0.2 mol/L NaCl	25°C		220 μmol photons $m^{-2} s^{-1}$	BG-11 medium			
62	Chrococcaceae	*Synechocystis*	*Synechocystis* sp. BASO511	609.8	mg/L	0.4 mol/L NaCl	25°C		220 μmol photons $m^{-2} s^{-1}$	BG-11 medium			
62	Chrococcaceae	*Synechocystis*	*Synechocystis* sp. BASO507	329.74	mg/L	0 mol/L NaCl	25°C		220 μmol photons $m^{-2} s^{-1}$	BG-11 medium			
62	Chrococcaceae	*Synechocystis*	*Synechocystis* sp. BASO507	339.54	mg/L	0.2 mol/L NaCl	25°C		220 μmol photons $m^{-2} s^{-1}$	BG-11 medium			
62	Chrococcaceae	*Synechocystis*	*Synechocystis* sp. BASO507	386.94	mg/L	0.4 mol/L NaCl	25°C		220 μmol photons $m^{-2} s^{-1}$	BG-11 medium			
63	Nostoc	*Nostoc*	*Nostoc* sp. PCC 7120	279	ug/g DW	0 mmol/L NaCl	30°C		30 μmol photons $m^{-2} s^{-1}$	BG-11 medium			
63	Nostoc	*Nostoc*	*Nostoc* sp. PCC 7120	650	ug/g DW	200 mmol/L NaCl	30°C		30 μmol photons $m^{-2} s^{-1}$	BG-11 medium			
63	Nostoc	*Nostoc*	*Nostoc* sp. HK-01	45	ug/g DW	0 mmol/L NaCl	30°C		30 μmol photons $m^{-2} s^{-1}$	BG-11 medium			
63	Nostoc	*Nostoc*	*Nostoc* sp. HK-01	177	ug/g DW	200 mmol/L NaCl	30°C		30 μmol photons $m^{-2} s^{-1}$	BG-11 medium			
64	Chrococcaceae	*Synechocystis*	*Microcystis aeruginosa* strain (FACHB-469)	0.16	pg/cell	0 mg/L $CaCl_2$	25°C		40 μmol photons $m^{-2} s^{-1}$	BG11 medium			
64	Chrococcaceae	*Synechocystis*	*Microcystis aeruginosa* strain (FACHB-469)	0.18	pg/cell	10 mg/L $CaCl_2$	25°C		40 μmol photons $m^{-2} s^{-1}$	BG11 medium			
64	Chrococcaceae	*Synechocystis*	*Microcystis aeruginosa* strain (FACHB-469)	0.23	pg/cell	20 mg/L $CaCl_2$	25°C		40 μmol photons $m^{-2} s^{-1}$	BG11 medium			
64	Chrococcaceae	*Synechocystis*	*Microcystis aeruginosa* strain (FACHB-469)	0.38	pg/cell	50 mg/L $CaCl_2$	25°C		40 μmol photons $m^{-2} s^{-1}$	BG11 medium			
64	Chrococcaceae	*Synechocystis*	*Microcystis aeruginosa* strain (FACHB-469)	0.44	pg/cell	100 mg/L $CaCl_2$	25°C		40 μmol photons $m^{-2} s^{-1}$	BG11 medium			
65	Chrococcaceae	*Synechocystis*	*Synechococcus elongatus* PCC 7942	25.87	ug/10^6 cells		28°C		50 μmol photons $m^{-2} s^{-1}$	BG-11 medium			7.5
65	Chrococcaceae	*Synechocystis*	*Synechococcus elongatus* PCC 7942	56.21	ug/10^6 cells	400 μM $MgSO_4 \cdot 7H_2O$	28°C		50 μmol photons $m^{-2} s^{-1}$	BG-11 medium			7.5
65	Chrococcaceae	*Synechocystis*	*Synechococcus elongatus* PCC 7942	57.1	ug/10^6 cells	2 μM $ZnSO_4 \cdot 7H_2O$	28°C		50 μmol photons $m^{-2} s^{-1}$	BG-11 medium			7.5
66	Oscillatoriaceae	*phormidium*	*Phormidium autumnale*	16.1	mg/g DW		20°C		70 μmol photons $m^{-2} s^{-1}$	BG11 medium			
66	Oscillatoriaceae	*phormidium*	*Phormidium autumnale*	29.3	mg/g DW	20 μg/L Cu	20°C		70 μmol photons $m^{-2} s^{-1}$	BG-11 medium			
66	Oscillatoriaceae	*phormidium*	*Phormidium autumnale*	26.6	mg/g DW	200 μg/L Cu	20°C		70 μmol photons $m^{-2} s^{-1}$	BG-11 medium			
66	Oscillatoriaceae	*phormidium*	*Phormidium autumnale*	22	mg/g DW	60 μg/L Zn	20°C		70 μmol photons $m^{-2} s^{-1}$	BG-11 medium			
66	Oscillatoriaceae	*phormidium*	*Phormidium autumnale*	22.8	mg/g DW	600 μg/L Zn	20°C		70 μmol photons $m^{-2} s^{-1}$	BG11 medium			
67	Nostoc	*Anabaena*	*Anabaena* sp. PCC 7120	47.67	mg/g DW	0 mM $CaCl_2$	28°C	cool-white fluorescent	65 μmol photons $m^{-2} s^{-1}$	BG11o medium (BG11 without $NaNO_3$)	shake-flasks	1000 mL	7.4
67	Nostoc	*Anabaena*	*Anabaena* sp. PCC 7120	41.79	mg/g DW	1 mM $CaCl_2$	28°C	cool-white fluorescent	65 μmol photons $m^{-2} s^{-1}$	BG11o medium (BG11 without $NaNO_3$)	shake-flasks	1000 mL	7.4
67	Nostoc	*Anabaena*	*Anabaena* sp. PCC 7120	76.27	mg/g DW	10 mM $CaCl_2$	28°C	cool-white fluorescent	65 μmol photons $m^{-2} s^{-1}$	BG11o medium (BG11 without $NaNO_3$)	shake-flasks	1000 mL	7.4
67	Nostoc	*Anabaena*	*Anabaena* sp. PCC 7120	30.03	mg/g DW	100 mM $CaCl_2$	28°C	cool-white fluorescent	65 μmol photons $m^{-2} s^{-1}$	BG11o medium (BG11 without $NaNO_3$)	shake-flasks	1000 mL	7.4
69	Nostoc	*Nostoc*	*Nostoc flagelliforme*	42.6255	mg/L	0% CO_2	25°C		60 μmol photons $m^{-2} s^{-1}$	BG11o medium (BG11 without $NaNO_3$)			
69	Nostoc	*Nostoc*	*Nostoc flagelliforme*	46.0149	mg/L	5% CO_2	25°C		60 μmol photons $m^{-2} s^{-1}$	BG11o medium (BG11 without $NaNO_3$)			
69	Nostoc	*Nostoc*	*Nostoc flagelliforme*	50.7462	mg/L	10% CO_2	25°C		60 μmol photons $m^{-2} s^{-1}$	BG11o medium (BG11 without $NaNO_3$)			
69	Nostoc	*Nostoc*	*Nostoc flagelliforme*	55.3098	mg/L	20% CO_2	25°C		60 μmol photons $m^{-2} s^{-1}$	BG11o medium (BG11 without $NaNO_3$)			
69	Nostoc	*Nostoc*	*Nostoc flagelliforme*	51.3164	mg/L	40% CO_2	25°C		60 μmol photons $m^{-2} s^{-1}$	BG11o medium (BG11 without $NaNO_3$)			
71	Chrococcaceae	*Gloeothece*	*Cyanobacterium Gloeothece* ATCC 27152	41		$NaNO_3$							
71	Chrococcaceae	*Gloeothece*	*Cyanobacterium Gloeothece* ATCC 27152	55.4		$NaNO_3$							
